# Transmembrane helical interactions in the CFTR channel pore

**DOI:** 10.1371/journal.pcbi.1005594

**Published:** 2017-06-22

**Authors:** Jhuma Das, Andrei A. Aleksandrov, Liying Cui, Lihua He, John R. Riordan, Nikolay V. Dokholyan

**Affiliations:** 1 Department of Biochemistry and Biophysics, University of North Carolina at Chapel Hill, Chapel Hill, North Carolina, United States of America; 2 Cystic Fibrosis Treatment and Research Center, University of North Carolina at Chapel Hill, Chapel Hill, North Carolina, United States of America; UNC Charlotte, UNITED STATES

## Abstract

Mutations in the Cystic Fibrosis Transmembrane Conductance Regulator (CFTR) gene affect CFTR protein biogenesis or its function as a chloride channel, resulting in dysregulation of epithelial fluid transport in the lung, pancreas and other organs in cystic fibrosis (CF). Development of pharmaceutical strategies to treat CF requires understanding of the mechanisms underlying channel function. However, incomplete 3D structural information on the unique ABC ion channel, CFTR, hinders elucidation of its functional mechanism and correction of cystic fibrosis causing mutants. Several CFTR homology models have been developed using bacterial ABC transporters as templates but these have low sequence similarity to CFTR and are not ion channels. Here, we refine an earlier model in an outward (OWF) and develop an inward (IWF) facing model employing an integrated experimental-molecular dynamics simulation (200 ns) approach. Our IWF structure agrees well with a recently solved cryo-EM structure of a CFTR IWF state. We utilize cysteine cross-linking to verify positions and orientations of residues within trans-membrane helices (TMHs) of the OWF conformation and to reconstruct a physiologically relevant pore structure. Comparison of pore profiles of the two conformations reveal a radius sufficient to permit passage of hydrated Cl^-^ ions in the OWF but not the IWF model. To identify structural determinants that distinguish the two conformations and possible rearrangements of TMHs within them responsible for channel gating, we perform cross-linking by bifunctional reagents of multiple predicted pairs of cysteines in TMH 6 and 12 and 6 and 9. To determine whether the effects of cross-linking on gating observed are the result of switching of the channel from open to close state, we also treat the same residue pairs with monofunctional reagents in separate experiments. Both types of reagents prevent ion currents indicating that pore blockage is primarily responsible.

## Author summary

Cystic Fibrosis is a severe genetic disorder caused by mutations in the gene coding for CFTR. This defect induces protein misfolding and channel dysfunction, resulting in the disruption of hydration of epithelial surfaces in different organs. Restoring channel activity in the mutant protein requires a greater understanding of mechanisms underlying correct channel function but has been obstructed by the lack of CFTR structural information. By combining experiments and computational techniques, we derive and validate structural models of CFTR in both the open and closed conformations that accurately represent an ion conduction pathway. We discover that restriction of the motions of pore lining transmembrane helices via cross-linking blocks ion currents. Our new structural models provide a platform for delineating CFTR gating mechanisms and identify therapeutic strategies for correcting CF defects.

## Introduction

Cystic Fibrosis (CF) is a fatal genetic disease caused by inheritance of mutations in the gene coding for CFTR. Medical advances in recent years have significantly extended the life span of CF patients, but the disease has no cure and is inevitably fatal. Delineation of the molecular mechanisms of channel gating and chloride ion permeation in wild type and disease-causing CFTR proteins is crucial for developing additional pharmaceutical strategies to restore physiological activity of the mutants.

Unraveling the molecular foundation of channel gating and ion permeation requires an accurate structural description of the CFTR channel. Several homology based CFTR models have been developed [1–10] employing available X-ray structures of ABC exporters as templates [11–16]. Data obtained from mutagenesis, labeling, cross-linking and single channel experiments suggest that some of the overall arrangement of membrane spanning helices is consistent with that predicted by the models. However, these homology models do not accurately reflect the structure of the channel pore [17] because of very low sequence similarity (<20%) in the membrane spanning domains between CFTR and other ABC proteins (for which crystal structures are resolved in their closed and open states). A recent report also provides evidence that CFTR possesses a gate near the middle of its ion conduction pathway, which is markedly different from well-known structures of ABC proteins having two separate gates located at the two ends of the pore, and with binding sites for the transported substrate [18].

In this study we develop inward and outward facing structural models of CFTR. As the outward-facing (OWF) state is believed to be the active state, we reconstruct the pore structure of this conformation by iteratively incorporating experimental constraints from cross-linking, labeling, mutagenesis, and single channel measurements using discrete molecular dynamics (DMD) [19,20] simulations that accurately represents an ion conduction pathway. Atomistic molecular dynamics (MD) simulations are carried out to generate the equilibrated structures of CFTR protein both in the closed and open configurations. Applying cysteine cross-linking and single channel measurements, we validate the equilibrated structure of the OWF state. After a 100 ns equilibration process, we determine the pore profiles of the CFTR channel using the 100 ns production run. In both conformations, these profiles match the general characteristics (i.e., wide extra- and intra-cellular vestibules connected via a narrow pore region) of the channel pore quite well. Our results reveal that, in the open state, the pore radius below the external channel entrance (*selectivity filter* formed by T1134, I1131 in TMH12 and S341, F337 in TMH6) is ~ 2.5 Å, similar to the radius of a hydrated chloride ion. In the closed state, the selectivity filter region is significantly narrower (< 1 Å). Comparison of the two conformations of the channel suggests that structural rearrangements of transmembrane helices (TMHs) 6, 9, and 12 play a central role in channel gating. Cross-linking of cysteine pairs introduced at specific positions in the helical pore region of TMHs 6, 9 and 12 provides a clear confirmation of the positioning and orientations of the pore-lining residues. The inhibitory effect of cross-linking of pairs of cysteine residues introduced at these predicted positions could either reflect the restriction of topological rearrangements of multiple TMHs involved in channel gating transitions or pore blockage by the cross-linking reagents.

## Results

### Molecular modeling of CFTR pore structures

To model CFTR pore structure in the putative conducting (OWF) and non-conducting (IWF) states, we apply an integrated computational-experimental approach. We use a simplified definition of the conducting/OWF/open and non-conducting/IWF/closed states and will use these terms interchangeably throughout the paper. We define the ‘conducting’ or ‘OWF’ or ‘open’ state to be the conformation in which channel pore is able conduct chloride ions from cytosolic to extracellular region and the pore dimension is large enough for the ion’s expulsion. Similarly, non-conducting or ‘IWF’ or ‘closed’ state is defined as the conformation in which the intracellular cavity is able to collect ions from the cytosolic domain, however the pore dimension is smaller at the extracellular end such that the channel blocks the passage of chloride ions. We construct the IWF state of CFTR using the homology based modeling method I-TASSER [17,21,22] (detailed description is provided in the supplemental information (SI), S1 Text, **Modeling inward facing state of CFTR** subsection). Next, we reconstruct our previous OWF atomistic model of CFTR [1] with special focus on the membrane domains by incorporating experimental constraints via DMD simulations. These constraints are taken from published functional data. Although the 3D structure of the permeation pathway(s) or pore(s) through which anions pass is not known, residues that influence ion conduction and span the entire pathway including membranous, extracellular and intracellular domains are considered pore-lining residues (S1 Table) [17,18,23–35]. The orientation of numerous residues in the TMHs 1, 3, 6, 9, 11 and 12 (known to participate in constituting the pore interior of the CFTR open channel conformation) of an OWF CFTR structure have been identified by various biophysical and biochemical experiments over the last decade as pore-lining and non-pore-lining residues. Our newly constructed OWF model is significantly more refined compared to the previous one and is validated versus a collective pool of functional data [17,23–30,36–44]. For instance, pore lining residues F200 and L218 in TMH3, W356, and D363 in TMH6, L986, T990, Q996, L997, and I1000, G1003 in TMH9 as well as T1134, S1149, D1152, and L1156 in TMH12 accurately orient toward the channel pore in the new structure (S1 Fig). Similarly, the correct orientations of non-pore lining residues are evident in the new model including several residues positioned in TMHs 1, 3, 6, 9, and 12 (e.g., G91, T94, L101, A198, L206, T351, P355, Y362, D984, P988, L1135, I1139, D1154, S1155, M1157, R1158, and V1160) [45]. Furthermore, our previous OWF model lacks the cytosolic extensions of TMH6 (extending from 366 to 390) and TMH12 (starting from 1156 to 1208). These fragments are built in our new OWF model. The same testing and modifications are not feasible for the IWF structure as the functional data relevant to the closed state discerning residue orientations in the membrane domain are unavailable.

Furthermore, structural comparison between the two models reveals that while some characteristics remain unaffected, orientations and positioning of several residues within the helices tracing the conduction pathway differ appreciably in the two structures. For example, TMH9 traversing the pore in the OWF state moves away from the conduction pathway especially in the lower transmembrane section in the IWF model (S1 Fig). This difference is evident from the altered relative positions and hence the interactions between the residues on TMHs 6 and 9 in the two structures (specific examples are discussed below). These subtle but fundamental topological rearrangements may be involved in transitioning from an active channel conformation to inactive ones and vice versa.

### Deriving optimized CFTR pore structures via all-atom MD simulations

**Fig 1:**
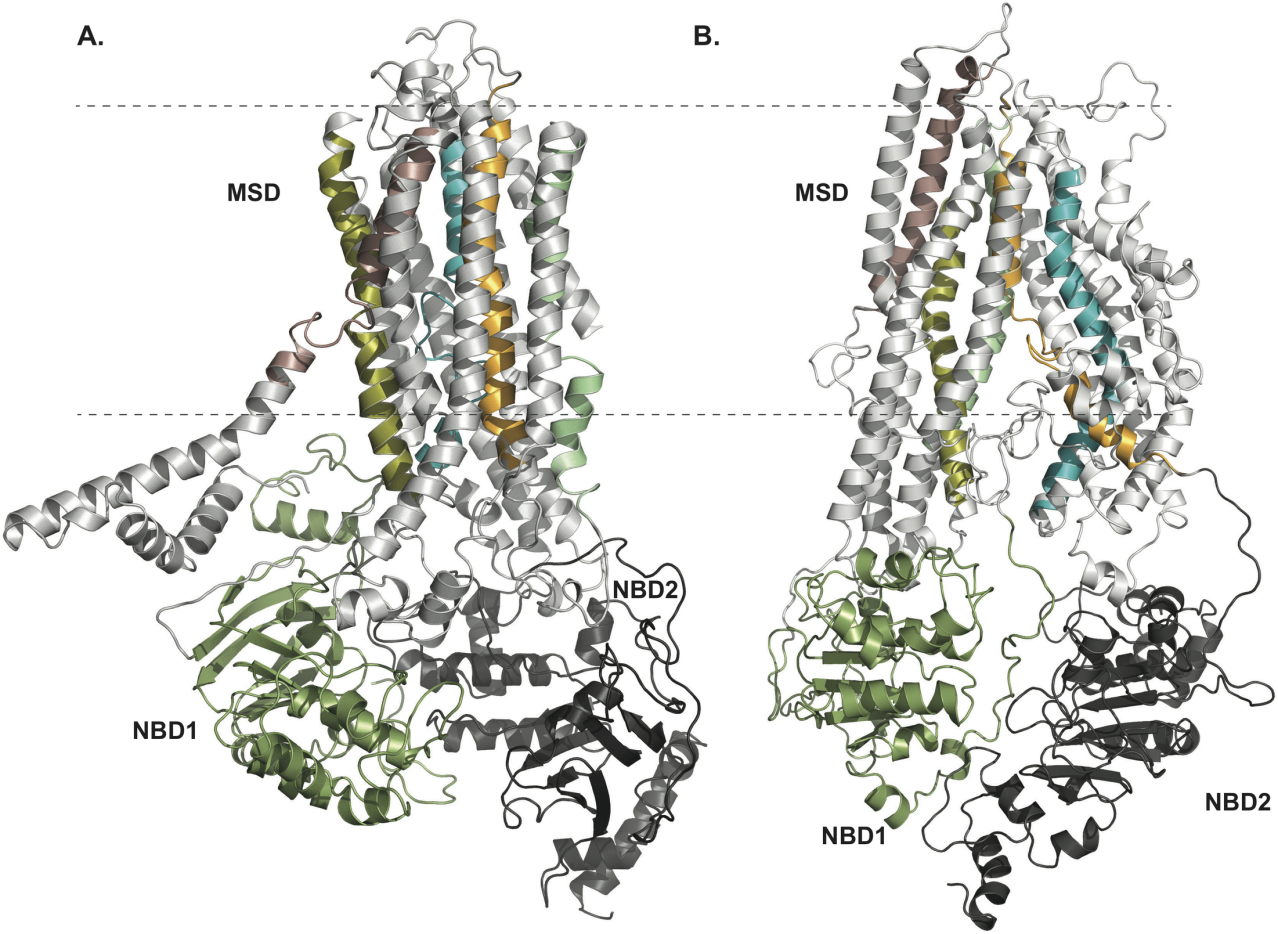
Refined pore structure of CFTR channel in open and closed conformations. (A) outward, and (B) inward facing structural models of CFTR as derived from our integrated experimental-computational method (see experimental procedures section). The R-domain, folded by an *ab initio* method by Serohijos et al.[1] is not depicted in these representations. TMHs 1 through 6 constitute membrane spanning domain 1 (MSD1) and TMHs 7 through 12 constitute MSD2. Different regions of CFTR structure are color-coded: MSD1 and 2 → white, NBD1 → dark green and NBD2 → dark gray. For visualizing the pore-lining helices in the 3D structure of CFTR, these are colored separately: TMH1: pink, TMH3: olive green, TMH6: cyan, TMH9: light green and TMH12: orange. The dashed lines represent the lipid membrane boundaries. To help position the TM helices in the CFTR sequence, different regions (i.e., twelve TMHs, NBD regions and R-domain) are labeled on the human CFTR sequence Aiming to regenerate experimentally verified structures of the putative pore region of CFTR in OWF conformations, this model is developed by implementing experimental restraints and DMD simulations. The structures of OWF and IWF states illustrated here are obtained from 200 ns long MD simulations (see also S1–S3 Figs, S10 and S11 Figs and S14 Fig).

The distinction between the previous and the newly developed OWF structures of CFTR and the diverse topological characteristics in both conformers presented above may be crucial for the physiological function of the CFTR channel. However, the conclusions drawn from the refined homology models of the CFTR channel in closed and open states could still be flawed as the static modeling might exert unphysical perturbations to the systems due to the introduction of experimental restraints. Furthermore, we risk over-fitting of the models through such modifications. Therefore, with the aim of exploring more energetically favorable, optimized structures of the channel in both open and closed conformations (Fig 1), we carry out 200 ns long all-atom molecular dynamics (MD) simulations using our starting OWF and IWF protein structures immersed into an environment mimicking native physiological conditions (detailed description of the MD protocol is reported in SI, S1 Text, **Molecular dynamics simulation protocol**). Each system reaches equilibration within the first 100 ns as determined from monitoring the root mean square deviation (RMSD) of the all atoms of the protein with time (S2 and S3 Figs).

**Fig 2:**
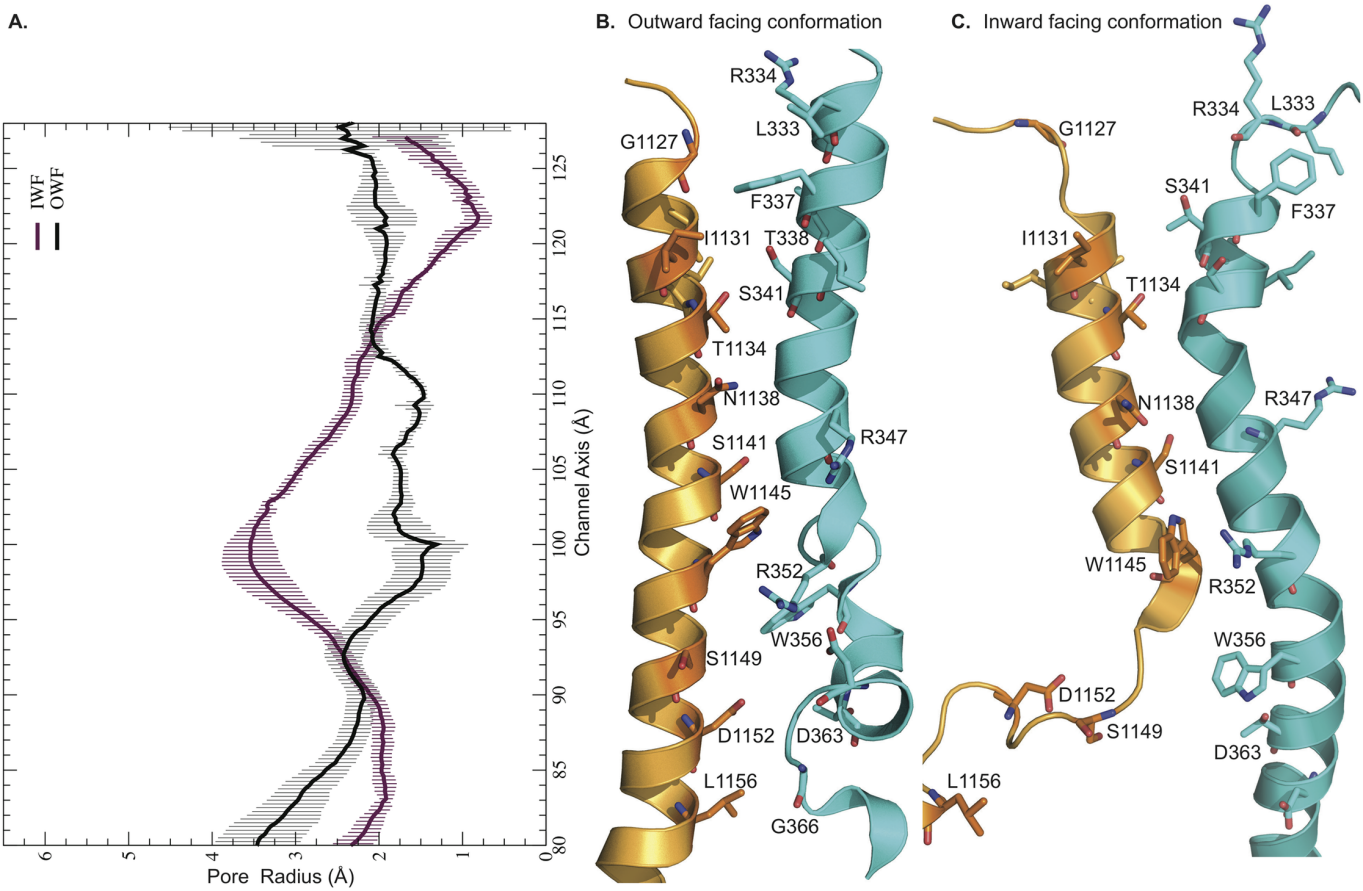
Pore profiles of CFTR channel in outward and inward-facing conformations. Relative positions of TMHs 6 and 12 from 200^th^ ns structures: A. Average pore profiles from corresponding MD trajectories, B. OWF, and C. IWF conformations. These radius profiles are calculated using 200 snapshots from the last 100 ns production run by applying HOLE software. Black and purple curves depict the pore profiles of the outward- and inward-facing states, respectively.

We determine the average pore profiles of the CFTR channel in two different conformations. The dynamic pore structures in the open and closed configurations show multiple unique features dissimilar from one another (Fig 2). For instance, the pore radius (R_pore_) at residue positions F337-S341 in TMH6 and G1127-I1131 in TMH12 is ~ 2.5 Å in the OWF state, while in the IWF state this region is completely closed off, yielding R_pore_ < 1 Å. This region has previously been identified as the selectivity filter of the CFTR protein [1]. Note that, the radius of a hydrated Cl^-^ ion is ~ 1.6–1.9 Å. Also, as expected, the pore radius pertaining to the region where the TM helices extend below the membrane boundary into the cytosolic region is considerably larger (R_pore_ ~ 3.0 Å) in the case of the IWF model after residue locations R352-W356 in TMH6 and W1145-S1149 in TMH12. This feature coincides very well with the hypothesis that the NBD regions dissociate in the closed channel thus creating a larger vestibule in the cytoplasmic ends of the membrane helices. We observe that both structures have large radii (R_pore_ ≥ 4 Å) at the two ends of the pore profile depicting the entrance to the extra- and intra-cellular vestibules. In the open channel, the equilibrium pore radius remains pretty constant with R_pore_ ranging between 2 and 3 Å ideal for permeating hydrated Cl^-^ ions.

### Testing model prediction using cysteine cross-linking

**Fig 3:**
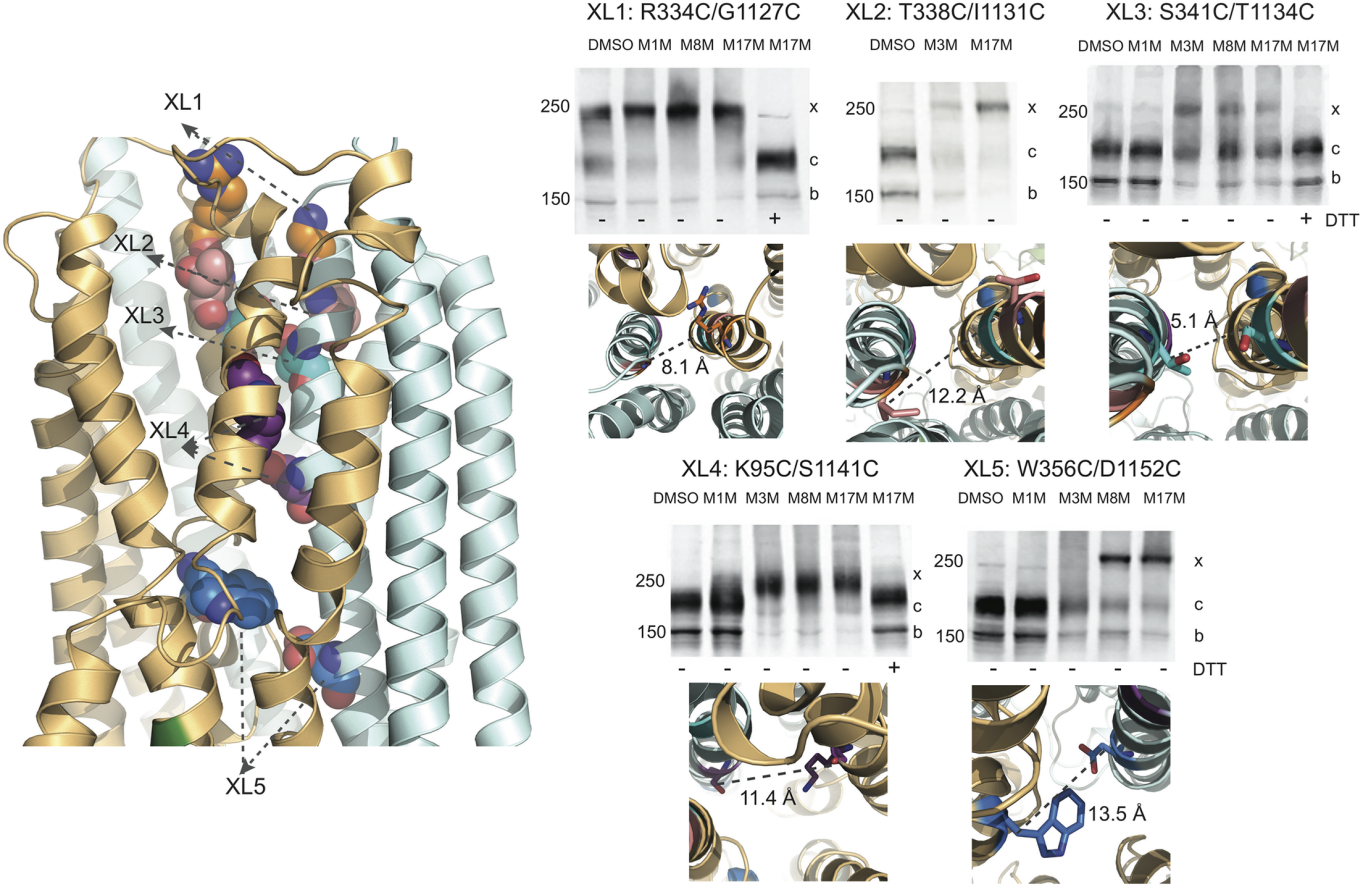
Cross-linking of cysteine pairs introduced in the putative CFTR Pore in outward facing state. Left: refined outward facing state of CFTR model exhibiting examples of residue pairs XL1, XL2, XL3, XL4 and XL5 (spheres) used to test the reliability of the modeled channel pore. Cysteine cross-linking experiments (protocol described in experimental procedures) are utilized for estimating the average distances between given residue pairs for model validation. MSD1: orange, MSD2: (cyan). Right: R334C/G1127C (XL1, top left) cross-links with ~2.1 Å (DMSO) < *d* < ~24.7 Å (M17M), T338C/I113IC (XL2, top middle) cross-links with *d* < ~24.7 Å (M17M), S341C/T1134C (XL3, top right) cross-links with ~6.5 Å (M3M) < *d* < ~24.7 Å (M8M), K95C/S1141C (XL4, bottom left) cross-links with ~6.5 Å (M3M) < *d* < ~24.7 Å (M17M), and W356C/D1152C (XL5, bottom right) cross-links with ~13 Å (M8M) < *d* < ~24.7 Å (M17M). The average distances between members of these pairs are indicated below the western blots. The outward facing CFTR structure presented here is obtained from 200 ns MD simulation. Immature core-glycosylated CFTR is marked as band b (near 150 KDa molecular weight marker); mature complex-glycosylated CFTR (band c); band x (just below 250 KDa molecular weight marker) represents cross-linked mature protein (see also S4 and S5 Figs).

To correctly determine the pore profiles for the IWF and OWF conformations, starting from the refined homology models, we estimate the average distances between the pore lining residues in various transmembrane helices from the equilibrium simulations. The mean distances between the residue pairs in TMHs 6 and 12 for both models are very similar in the membraneous domain, while most divergences are observed in the TMH extensions into the extracellular and cytoplasmic regions (S2 Table). On the other hand, the relative distance between the residue pairs in the TMHs 3 and 9 as well as in TMHs 6 and 9 are appreciably different due to the altered positioning of TMH9 in the two structures. This apparent translational movement and rotational alteration exhibited by TMH9 (S1 Fig) between the two structures remains unperturbed even after the 200 ns equilibration, suggesting that the positional shift of TMH9 might be a hallmark feature distinguishing the two conformations. To test the validity of the newly derived pore structures in both conformations, we have relied primarily on the cross-linking of cysteine pairs introduced into a functional Cys-less CFTR construct [1]. These experiments also provide a basis for further model refinements. We have previously utilized cysteine cross-linking to successfully model the crucial interfaces between the cytoplasmic and membrane domains (e.g. NBD1-CL4 and NBD2-CL2) of the CFTR molecule [46] and studied their interactions, but the technique has not yet been extensively used to test predicted relationships between TMHs. Others have employed functional assays [4,30,42,47,48] to indirectly determine the relative positions of CFTR residues without biochemical confirmation of the extent of cross-linking. Here, we perform cysteine cross-linking experiments on 29 residue pairs spanning the membrane domains to characterize the spatial arrangements of the TM helices constructing the pore interior. Among all tested pairs, 23 of them show cross-linking while six do not. With all cross-linked pairs, only the mature form (band c) and not the immature form (band b) of CFTR is cross-linked as shown by the appearance of the band with retarded mobility (x band), confirming that the residues are cross-linked by MTS reagents only when the protein has achieved a native conformation (Fig 3, S4, S7 and S8 Figs). While not reliable in the dynamic, disordered regions, cysteine cross-linking results are especially informative in determining the accurate distance range between two cysteine substituted residues in the pore, where the fluctuation of membrane helices are significantly limited due to the viscous nature of the membrane and therefore can be used to create a two-dimensional map for both testing and refining the structural features of the CFTR pore. For instance, the cross-linking of S341 and T1134 clearly shows that the c band is converted to the x species most efficiently by M3M, and partially by M8M and M17M (Fig 3). This finding suggests that the S341 and T1134 reside within a distance of ~ 6.5 Å. Indeed, the average distance between the two residues in the current OWF and IWF structural models are 5.1 and 5.6 Å, respectively. The partial cross-linking of S341C/T1134C by M8M and M17M occurs because while these reagents are much longer in fully extended form; their flexible nature allows them to connect residues with shorter separation. Another example is W356C/D1152C; the full conversion of the c to x band of this species happens only upon the administration of M8M and M17M (Fig 3). Our models show that the relative average distances between the Cβ atoms of these residues, 13.5 Å (OWF model) and 18.9 Å (IWF model), are in good agreement with the relative positioning of pore lining residues obtained from cysteine cross-linking. This result indicates that under functional conditions, the distances separating several residue pairs in the membrane-solvated regions are similar in both conformations. Hence, the cross-linkers can identify either conformation or both if the relative separation between two cysteine-substituted residues is within effective cross-linking range. Similarly, according to the IWF and OWF models, the inter-distance of T338C/I1131C is ~ 9.4 Å and 12.2 Å (Fig 3 and S5 Fig), respectively, which explains only a partial cross-linking of this residue pair by M3M and full cross-linking by M17M (Fig 3). The residue pair K190C/K978C at the cytoplasmic ends of TMHs 3 and 9 are 32 Å apart in the open conformation, and ~ 19 Å in the closed one (S2 Table and S4 Fig). Thus, it is possible that M17M only cross-links the closed species, which explains the presence of complete cross-linking of the K190C/K978C species only when treated with the longest reagent. Similarly, the complete cross-linking of the R334C/G1127C species appears upon treatment with M8M. Note that their separation distance is 8 Å and 14 Å in the open and closed states, respectively; shorter cross-linkers are only minimally effective. It should also be mentioned that R334 and G1127 are located at the extracellular ends of TMHs 6 and 12 and are highly dynamic. On the other hand, K95 and S1141 are separated by 12 Å in the OWF structure and most likely only this conformation is cross-linked with MTS reagents. The distance between these residues is ~ 25 Å in the IWF conformation, which is beyond the largest distance that can be cross-linked by the longest cross-linker (M17M) used in our experiments.

Taken together, among all the cysteine pairs tested, 23 pairs are cross-linked (S2 Table) by MTS reagents. The average relative distances between cysteine-substituted residue pairs retrieved from the channel pore of the equilibrated IWF and OWF structures and the cross-linking experiments correspond very well (S2 Table, Fig 3, and S4 and S5 Figs). An additional six pairs (S3 Table), used as controls, do not exhibit cross-linking as predicted by the models. As visualized from the models, among all the residue pairs in this category, either one of the two pairing residues (e.g., F342, T339, T338 in TMH6 and L188 in TMH3) is oriented away from the pore interior and/or the separation distances between the additional residue pairs (e.g., R334 and V1010; L188 and S185 with V1163) are longer than the longest MTS reagent used in our experiments. The overall agreement between the relative positions/orientations of the partnering residues as identified from the models and the cysteine cross-linking results (S2 and S3 Tables) supports the fidelity of our new models.

### Functional relevance of inter-helical contacts in the transmembrane domain

**Fig 4:**
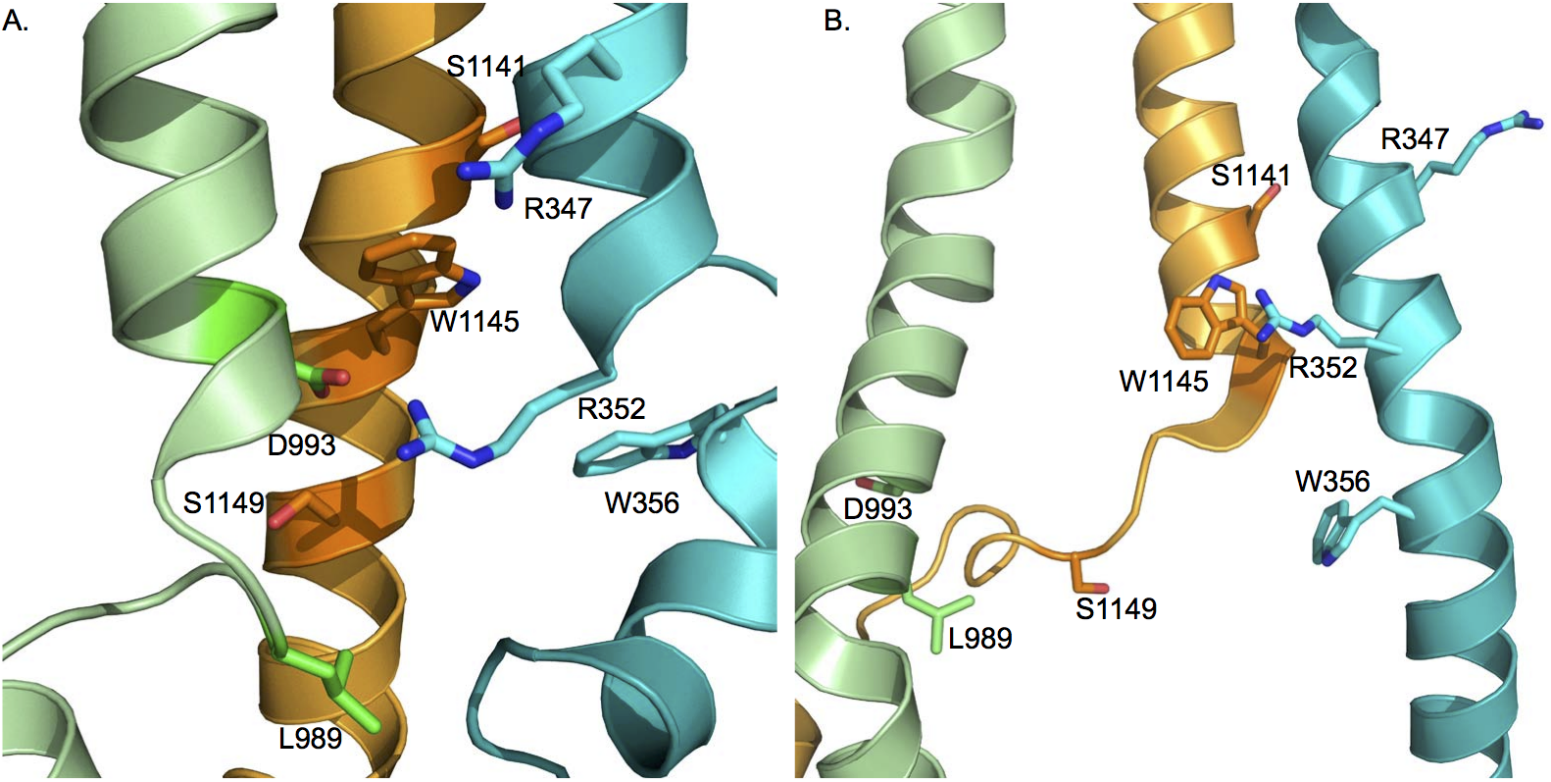
Relative positions and orientations of selected pore lining residues on membrane helices 6, 9 and 12 used to test functional effects of their cross-linking. A. outward-, and B. inward-facing states. TMH6, TMH9 and TMH12 are shown in cyan, green and orange cartoon representations, respectively (see also S1 and S9 Figs).

**Fig 5:**
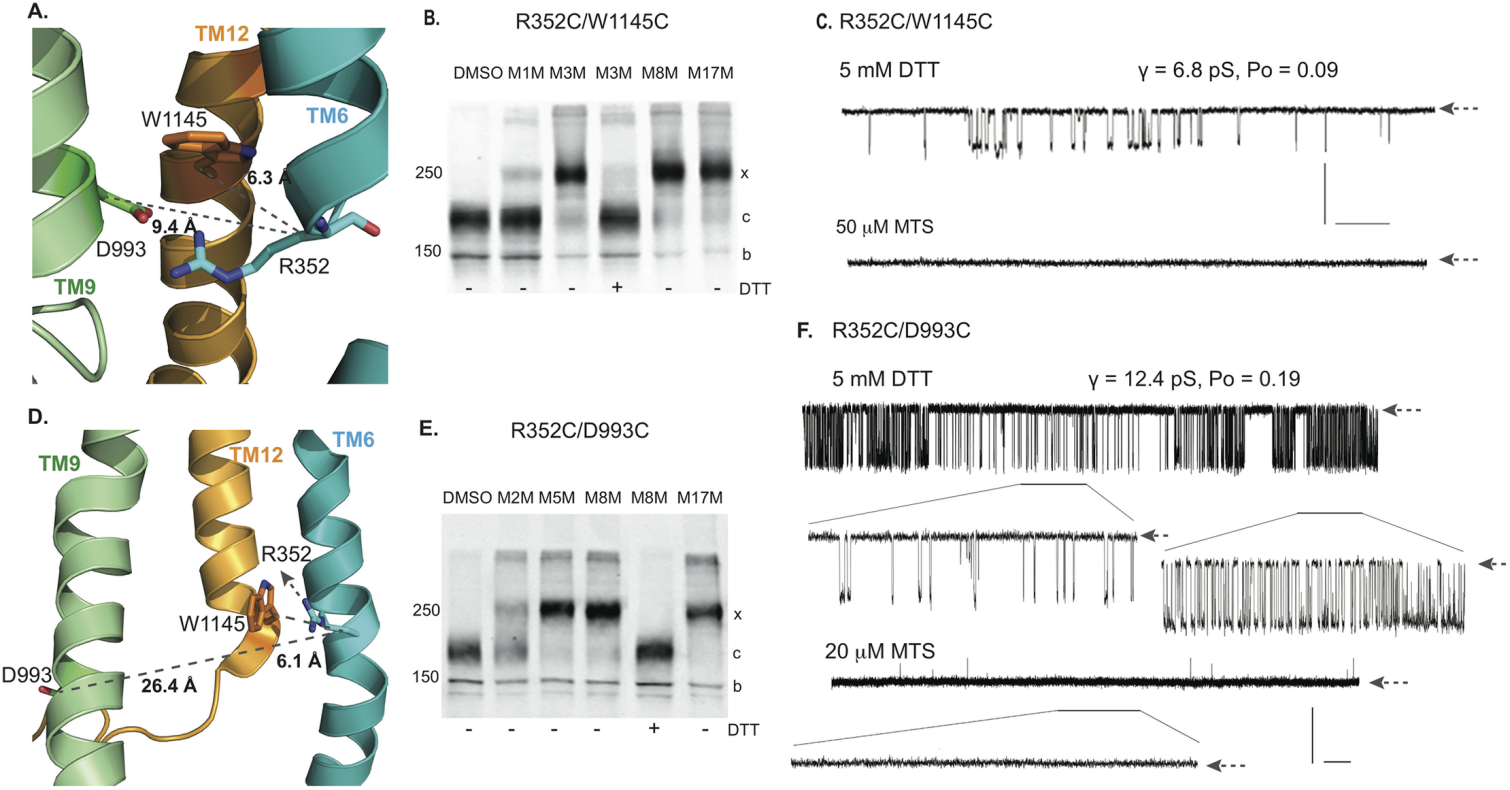
Cross-linking of membrane spanning helices 6, 9 and 12. A. Relative positions of R352 in TMH6, D993 in TMH9, W1145 in TMH12 in outward facing state. B. Western blot of Cys-less CFTR R352C/W1145C before (lane 1) and after treatment with 20 μM of MTS cross linkers (lanes 2, 3). No change in mature band (c) position before treatment indicates that there is no spontaneous disulfide bond formation between R352C and W1145C while M3M, M8M and M17M treatment cause a strong shift of the c band to the higher molecular weight band cross-linked form (x band). C. Single channel recordings showing γ = 6.8 pS under reducing conditions (DTT; upper tracing) or non-conductive state as a result of cross-linking by 50 μM M3M (lower tracing). D. Positions of R352, D993 and W1145 in the inward facing state. E. Western blot of Cys-less CFTR R352C/D993C before (lane 1) and after treatment with MTS (20 μM) cross linkers (lanes 2–4). No c band appears without cross-linker addition indicating that a spontaneous disulfide bond is not formed. F. 10 min single channel recording under reducing conditions (DTT; upper tracing). Two segments with different gating kinetics and Po but identical γ = 12.4 pS with extended time scale are labeled by bars and shown below the upper trace in the extended time scale. No opening is observed after addition of 20 μM M5M reagent from the “cis” side (middle tracing). Blank recording of 60s segment in extended time scale (lower tracing). Single channel closed states are shown by arrows at the right end of each trace. Vertical (Y-axis) scale bar represents 1pA for all traces is shown on right. Horizontal (X-axis) scale bars are different for each trace: 30s for the topmost trace and 10s for the bottom traces (see also S6–S8 Figs).

The cross-linking of the cysteine pairs outlined above supports the relative positioning and orientation of residues in different parts of the membrane spanning portions of CFTR in the computational models. To assess the functional impact of cross-linking or modifying cysteines at some of these positions we focus on helices 6, 9 and 12 already reported to make crucial contributions to the CFTR pore [6,8,17] (Fig 4). For example, the positively charged R352 residue near the cytoplasmic end of TM 6 appears oriented towards and within ~ 6 Å of W1145 in TM12 (Fig 5A and 5D). This relationship is confirmed biochemically by the essentially complete cross-linking by the MTS reagent, M3M as well as by the longer compounds, M8M and M17M but with minimal cross-linking by the shorter, M1M (Fig 5B). This R352C/W1145C construct exhibits low open probability channel activity with a unitary conductance approximately half that of wild type as expected [42] due to removal of the R352 positive charge (Fig 5C, upper tracing). On addition of M3M, gating was entirely arrested (Fig 5C, lower tracing). Since the relationship between the residues at these positions is little changed as visualized in the OWF (Fig 5A) and IWF (Fig 5D) models, the inhibition of gating presumably is not due to a shift between these two states. To test the alternative possibility that the pore is simply blocked when spanned by the MTS reagent, the influence of the same bifunctional compounds on the single cysteine W1145C variant is examined (S6A Fig). Even in the case of MTS compound binding to only one member of this cysteine pair results in inhibition of gating, thereby supporting the notion of pore blockage rather than structural transition of the channel from conducting to non-conducting state.

The interaction of the R352 residue with an oppositely charged residue, D993 in TM9 has been extensively characterized by McCarty and colleagues [32,42,43] who provided evidence that the amino acids can form a salt bridge, perhaps during channel opening. As viewed in our outward facing model (Fig 5A) R352 appears to be as accessible to D993 as to W1145, whereas in the IWF configuration (Fig 5D) the D993 containing portion of TM9 appears to have moved with D993 turning away from the pore, and becoming inaccessible to R352. When an R352C/D993C construct is treated with bifunctional MTS reagents, the pair is completely cross-linked by either M5M or M8M (Fig 5E), suggesting that their binding occurs in the OWF state that may coincide with a channel open state. However, addition of an effective MTS cross-linker does not promote a channel open state (Fig 5F). On the contrary, the robust full conductance gating observed under reducing conditions (Fig 5F, upper tracing) is entirely inhibited by the cross-linker (Fig 5F, lower tracing). This result, similar to that with the R352C/W1145C pair would not be surprising if the binding of either bifunctional or monofunctional reagents to these pore lining residues could block the ion passage and indeed we find that this is the case. The M3M cross-linker inhibits the single cysteine D993C variant (S6B Fig) and the monofunctional MTSBn blocks the R352/D993C channels (S6C Fig). These findings do not conflict with the demonstrated ability of the residues normally present in these positions to form a salt bridge (Discussion). However, the demonstration that a cysteine replacing R352 is efficiently cross-linked to either W1145C or D993C combined with the prevention of ion permeation by the binding of mono- or bi-functional MTS reagents provides strong support for the modeled positioning and orientation of these residues within the pore.

The ability of cysteines replacing residues at other positions in TMH 6 and either TMH 9 or TMH 12 were similarly evaluated biochemically and their impact on single channel gating determined. First, we observe that a cysteine substituted for R347, a more externally located TMH 6 residue, also known to strongly influence channel conductance [26,27,44], could be cross-linked to D993C in TMH 9 by longer MTS reagents (M8M and M17M) and to S1141C in TMH 12 by M5M (S7B and S7E Fig). Under reducing conditions prior to cross-linking both of these pairs of constructs had low conductance (4.1 pS) single channel currents due to the absence of the R347 positive charge and in both cases the currents are entirely ablated by exposure to MTS reagents (S7C and S7E Fig). Interestingly, the reduced channel conductance of the R347C/D993C and R347C/S1141C pairs is the same indicating that the absence of the R347 positive charge has the same impact when removed together with an oppositely charged (D993) or an uncharged (S1141) amino acid. This result contrasts that with the R352/D993 salt-bridging pair (Fig 5F) and suggests that R347 may not form a salt bridge with D993.

Pairs of uncharged residues located further towards the cytoplasmic ends of these helices also are replaced with cysteines and similarly analyzed. W356C of TMH 6 is completely cross-linked to S1149C in TMH 12 by M8M and M17M (S8C Fig) and to L989 in TMH 9 (S8D Fig) by shorter cross-linkers. The full conductance gating displayed by these constructs before cross-linking is completely inhibited after cross-linking as with the other TMH 6/12 and TMH 6/9 cysteine pairs (S8E and S8F Fig). Thus, the predicted arrangement of residues at different depths of the membrane embedded portions of these three pore-lining helices is supported by the capacity of cysteines substituted at these locations to be cross-linked and by the inhibition of channel activity by their binding of either bi-functional or mono-functional sulfhydryl binding reagents.

## Discussion

### Modeling CFTR pore structures through integrated experimental-computational protocol

CFTR is the only member of the ABC protein super-family that acts as ion channel rather than a transporter of organic molecules indicating that some aspects of its permeation pathway and gating mechanism must be distinct. Furthermore, a number of pathogenic mutations associated with CF are located in the CFTR pore region and affect channel gating. Therefore, with the objective of developing a platform that can not only serve to understand the role of the membrane domains of this protein in the gating mechanism, but also other structural and functional aspects and drug discovery projects, we have modeled OWF and IWF states of CFTR. Upon building the CFTR structures in both conducting and non-conducting states (that is consistent with a collective pool of published functional data) [17,23–30,36–44], we have rigorously validated the structural models utilizing the cross-linking of cysteine pairs introduced at model predicted positions (data reported in Figs 3 and 5 and S4, S5, S7 and S8 Figs, as well as in S2 and S3 Tables).

To verify our model predictions, we rely on cysteine cross-linking since this technique is more effective than the single cysteine accessibility scanning (SCAM) technique because it provides a more precise two-dimensional map of the pore by cross-linking residue pairs of varying separation distances. When treated with MTS reagents, if they are within the pore, their functional impact can be detected with single channel measurements thus confirming the locations as well as the relative positions of the partnering residues within the pore. SCAM alone, on the other hand, can yield ambiguous data in determining whether the residue is pore lining or not. For instance, in previous work Dawson et al. [6] reported that several consecutive residues within the TMHs 6 and 12 are pore lining, but in the pore region the amino acids presumably assume helical structure. Therefore, five or six consecutive residues cannot all be pore lining or non-pore lining. This result indicates that those reagents may interact non-specifically or might modify the protein structure significantly upon interaction, which can lead to ambiguous interpretations.

In addition, we infer from our single channel measurements of cysteine cross-linked constructs that, like other ABCC proteins, CFTR exists in various closed and open states that are not structurally strikingly distinct from one another. Therefore, cysteine cross-linking can identify several or at least a few specific configurations, when the residue pairs in question are within the interaction range of the MTS reagents used. In fact, recent cryo-EM studies of the P-glycoprotein protein and MsbA transporters reported that, in a cellular environment the overall population of these kinds of proteins is an equilibrium mixture of myriads of open and closed states [49,50]. Our results also demonstrate that cross-linking could be used to arrest the CFTR protein in various stable channel conformations and thereby may aid structural determination by crystallography and electron microscopy.

A recent computer modeling study [10] also reveals a similar pore structure to that seen in our novel CFTR open pore structure resulting from MD simulation (that identifies F337 and the subsequent residues S341, M348, and R352 in the TMH6 and the TMH12 residues T1134, N1138, S1141 and W1145 as pore lining residues, S1 Fig). Furthermore, by comparing our newly reconstructed models in the IWF and OWF conformations, we observe that structural reorganization between the TMHs 1, 6 and 12 (residues 102–106, 337–341 and 1134–1138) in the channel pore induces the transition between the conducting and non-conducting states (S9 Fig). This narrow region is believed to form the selectivity filter thus facilitating the specific ion permeation in channel open state. The overall construct of this so-called selectivity filter region found in our study matches that in the CFTR model reported by Corradi et al. [10].

### Structural comparison of IWF model with newly solved cryo-EM structure of CFTR

During the course of the review process of our paper, Zhang et al. [51] published the cryo-EM structure of the human (PDBID: 5UAK) and zebrafish (PDBID: 5TSI) CFTR proteins in its’ inactive (closed) state. We compare our homology-based IWF model and the MD-generated model with the cryo-EM based structure and the overall match is very good, close to the resolution of the cryo-EM structure (S10 and S11 Figs and S4 and S5 Figs). Overall, the RMSD values indicate good correspondence between all structures. Similar structural deviations are observed when comparing our models versus the zebrafish CFTR (PDBID: 5TSI). This is not surprising as RMSD between the human and zebrafish CFTR in PDB are within 1.7 Å and the resolution of both structures are ~ 4 Å. For the MSDs and the NBDs, the corresponding RMSDs are slightly higher than the resolution of the published cryo-EM structure since in those cases we also take into account unstructured regions such as intracellular-, extracellular-loops, and other connecting domains. These differences are a little higher when comparing the cryo-EM structure versus the MD structure. This discrepancy may be due to the different conditions used in the MD simulations than the ones used in cryo-EM experiments thereby yielding an alternative structure but equally probable average state. It is also possible that these deviations are mainly due to unstructured domains. There is no structural information available for the rational modeling of the unstructured domains.

Specifically, the RMSDs of TMHs 7, 8 and 12 are significantly larger than other TM helices. For example, we do not observe the kink on TMH8 reported by Zhang et al. [51]. TMH12, in our model, while positioning itself in relatively the same orientation and angular position, show a noticeable α-helical unfolding from residue A1146 to S1155 in the IWF model. It remains to be determined whether these apparent differences between our models versus the cryo-EM structure, i.e., the unfolding of TMH12 or the absence of the kink in TMH8, reflect functionally relevant different states, the limited resolution of the cryo-EM structure (~ 4.0 Å) or imprecision of the predicted structure. The cryo-EM structure represents one of the many different states that CFTR channel can assume under physiological conditions. The unfolded region is basically the cytoplasmic extension of TMH12 and is exposed to solvent, that might alter its’ structure considerably. The condition at which the cryo-EM structure is solved also requires several specific constraints. Our MD derived model may depict an alternative, slightly different conformation. Regarding the modeling of TMH7, it may be possible that incorrect positioning of the disordered R-domain might have altered the orientation of the helix significantly and therefore yield higher RMSD.

The relative accessibility of various residues from the intra-cellular or extra-cellular domains as well as the pore lining residues can be assessed in our model structure versus the functional data reported in the literature and the recent cryo-EM model of the zebrafish CFTR model (PDBID: 5TSI) [51]. The residue positions in both the homology, and MD-derived models compare fairly well to the published structure (S12 Fig) thus confirming the reliability of our structural model. For instance, the residues accessible from the intracellular side (S307, F311, V345, M348, A349, R352, Q353, T1112, S1141, T1142, Q1144, W1145, V1147, N1148, S1149); residues accessible from the extracellular side of the membrane (R104, L323, A326, R334, K335, I336, I1121, T1122, I1131, and I1132) and the pore lining residues (Q98, P99, L102, F337, T338, S341, I344, T1115, S1118, N1138, and M1140) match perfectly with previous experimental data (S12 Fig).

Both the cysteine cross-links tested in this study (that includes pore region) and our previous work (addressing the inter-residue distances between the intracellular and NBD regions) are mapped onto our newly developed closed states of CFTR (S4 and S5 Tables). For the majority of residue pairs in the CFTR pore, the distances retrieved from the cross-linking experiments versus the structural model are very close (S4 Table). In fact, the estimated distances between the MD-generated model and the cryo-EM structures show more similarities in many cases (e.g., S341C/T1134C, R352C/Q996C, K190C/K978C, N186C/K978C) than the ones directly obtained from homology modeling. Therefore, we can conclude that MD simulation further optimizes the global arrangement of the preliminary homology model. The few exceptions where the cross-linking data diverge significantly from our models as well as the cryo-EM structures involve a number of residue pairs in TMHs 6 and 9 (e.g., R347C/D993C, R352C/D993C).

In addition to the pore profiles of our IWF models, the relative positions of the NBD regions as well as their locations with respect to the intracellular loops align well with the cryo-EM structure [51]. The asymmetric opening at the NBD interface is also preserved in our models as seen in the cryo-EM structure (S13 Fig). This asymmetry is believed to exist due to the presence of the R-domain being located on the side of the degenerate site [51]. It is true that, there are some deviations between the inter-residue distances estimated from all the IWF models and the ones derived from cross-linking experiments. A possible explanation for this discrepancy could stem from the fact that the cross-linkers most likely capture the OWF conformations more frequently than the IWF ones. In fact, cross-linking results for these pairs resemble quite well the OWF conformation (S2 Table). In cells, CFTR protein exists in many different stages of activation and it is hard to arrest one conformation versus the other. Also, it is possible that the channel open/close configurations are, in fact, an ensemble of very many states with small structural differences.

### Dynamic alterations in the CFTR channel is essential for gating

Intriguingly, by comparing our open and closed models we find that there are significant conformational rearrangements of TMHs 6, 9 and 12 between the two structures. In line with previous studies, our results demonstrate that these structural alterations may be responsible for transitions between channel open and closed states, which could arise from translational and rotational movements of several TMHs [52,53]. We examine this hypothesis by cross-linking cysteine pairs in the helical pore region of TMHs 6, 9 and 12 (Fig 5 and S7 and S8 Figs). Our inspection provides a clear validation of the positioning and orientations of the tested residues (R347, R352, W356 in TMH 6; D993 and L989 in TMH 9; as well as S1141, W1145 and S1149 in TMH 12) within these TMHs. However, these data do not definitively establish that helical movements are involved in channel gating because the MTS reagents are found to occlude the channel pore. In fact, modifications of single cysteine substitutions of residues engaged in interactions between helices, with monofunctional reagents show that the prevention of ion permeation in our single channel measurements can arise because of the obstruction of ion passage by these molecules (S6A and S6B Fig). However, this finding does not preclude the possibility that restricting helical motions by the formation of cross-links between them may also contribute to the changes in conductance observed.

In addition to multiple structural features that are distinct in the IWF and OWF models (as discussed above), the spatial relationship between the salt bridge forming R352 and D993 is of particular interest. These two residues are oriented directly towards each other in the OWF state (Fig 5A). In the IWF state, however, their association appears not to be possible as the D993 portion of TMH 9 seems to rotate away from the pore obstructing its accessibility to R352 in TMH 6 (Fig 5D). McCarty and colleagues [43] provided evidence that R352 and D993 interact electrostatically and indicated that the cross-linking of cysteines at these positions might support a channel open state. To test this proposal, we assess the possibility of cross-linking of R352 and D993 (Fig 5E and 5F). The mature form of this construct is strongly cross-linked by M5M, M8M and M17M but only partially by the shorter M2M (Fig 5E) that the McCarty group had employed [43]. Contrary to their findings, we observe only a closed channel state of regardless of whether the R352C/D993C channels are treated with monofunctional or bifunctional cross-linkers (Fig 5 and S6C Fig); no locked open channel is detected. This difference could be due to the fact that our cysteine cross-linking experiments are carried out in a Cys-less background, which ensures the interactions of MTS reagents only with the intended residue positions. McCarty et al. [43] performed their experiments using wild type CFTR containing 18 native cysteines. Therefore, in addition to the targeted R352C/D993C pair, there may have been modifications of other cysteines. Earlier studies showed that chemical modification of native cysteine residues in the R-domain stabilize a channel open state [54] in the wild type protein, which might be mistakenly interpreted as arising from cross-linking between R352 and D993. With the objective of finding structural elements that distinguish active and inactive conformations, we find that translational and rotational changes in TMH9 seem to be crucial in breaking the salt bridge between R352 and D993 residues that is a hallmark feature of the open state.

The traditional conviction regarding CFTR gating requires that the NBDs are separated in the IWF state, and in the OWF state NBDs form a tight sandwiched configuration upon phosphorylation and nucleotide binding. Our equilibrated OWF state shows that the channel can be in conductive state even when the nucleotides are not bound to the NBDs. For example, the distance between the S549 and T1246 in Walker A and B motifs in our model is slightly larger, ~ 12 Å (S6 Table) compared to other models ~ 8–9 Å. Other residues within the vicinity of NBD interfaces also depict slightly larger opening between the two NBDs than seen previously. While nucleotides are not added in this set of simulations with the OWF state, the relative positions of NBDs as well as the dimerized NBD interface has been maintained during the length of the simulations. To validate our claim, we have calculated the relative average distances between the Cβ atoms of several residue pairs within the NBD interface throughout 200 ns long simulations, which were previously reported [55]. From the small standard deviation in the relative distances for all pairs, one can conclude that the dimerized interface is stable during the course of equilibration in both OWF and IWF model (S13A Fig). Certainly, this differential feature in our model, compared to existing ones, yields a small rearrangement of the coupling helices between the TMHs 8 and 9, as well as the TMHs 11 and 12 and creates an intracellular vestibule. While tight contact between NBD1 and 2 are the hallmark of currently published structures, our observation affirms that morphing from channel ‘closed’ to ‘open’ state for ion permeation may not require major conformational change. Instead, small structural alterations may be enough for transitioning between these two states. From our data, we conclude that indeed CFTR channel is dynamic and can exist in many alternative open and closed structures and rather than one specific ‘conducting’ and ‘non-conducting’ conformations.

While elucidation of the CFTR channel gating mechanism remains incomplete, accumulated experimental evidence indicates that the channel is highly dynamic and can access more than one or two specific states that could be designated as open or closed states [42,56]. Indeed, our single channel measurements distinguish multiple channels with different channel open probabilities (S8 Fig). These transitions may not require significant structural shifts such as the dissociation of NBDs and can be achieved by subtle topological realignment of various pore forming membrane helices. In reality, the energetics and structural landscape of CFTR is very complex and small spatial modifications in the membrane helices can give rise to multiple transient intermediate states. In fact, based on other MD simulation studies, Mornon et al. [9] and Corradi et al. [10] suggested that CFTR could adopt various conformations that could be referred as “open” and “closed” conformations. These transition states result from minor topological reorganization of the transmembrane helices. However, their conclusions were based on very short simulations (10–30 ns long). From the cluster analyses of 1000 snapshots of the protein (acquired from our 100 ns production runs after equilibration) for both IWF and OWF models, we do not observe states resembling different conductive and non-conductive states (example OWF states shown in S14 Fig). As shown in the RMSD plots presented for both closed and open states, indeed the systems require long times for equilibration (~100 ns). While identifying the fine structural determinant characteristics of these alternative conducting and non-conducting conformations are imperative to understand CFTR gating, long time molecular dynamic studies are required. We can also gain insights from developing CFTR structures by applying different ABCC proteins as structural templates for which crystal structures have been solved at different states (nucleotide-bound and -unbound) [57]. To this end, it should be noted that, in the cell, transitions between different conductive and non-conductive states are shown to occur in the hundreds of millisecond time scale. Therefore, drawing conclusions based on only tens of nanoseconds may be erroneous, as the results might be influenced by non-equilibrium, unphysical forces and the identified states may not be real, and thermodynamically stable. To unveil the atomistic structures of such physically tangible, intermediate states visited by the CFTR molecule and the molecular underpinnings of these transformations, at least microsecond(s) long simulations are required. For example, such simulations may be able to illuminate the structures of the recently reported transitory channel open states [58]. Structural characterization of these intermediate states and insight into the dynamics of the opening conformational change will facilitate understanding of the gating defect in ΔF508 CFTR.

In summary, by combining experiments and computational techniques, we derive structural models of the CFTR in both the open and closed conformations that accurately represent structural and dynamic features of the ion conduction pathway. Our data suggest that structural alterations of TMHs may also generate multiple unique channel conductance states. While our study emphasizes the importance of membrane helices in the channel gating regulation, more detailed molecular level understanding of how these helices control this process remains to be elucidated. By reconstructing and validating the pore region of the OWF state of CFTR through the implementation of experimental information in the active conformation, we present a platform to understanding the molecular mechanisms involved in the CFTR function. Additionally, these models could be useful in determining the structural and dynamic deviations pertaining to the disease states (especially the mutations that affect channel conductance and gating mechanisms). This information may prove essential in designing extended personalized medicine for CF.

## Materials and methods

### Computational-experimental approach to derive structural models of CFTR

The putative pore region of the OWF state of our previous CFTR model [1] is refined by iteratively implementing experimental constraints from a large body of functional data (e.g., cysteine scanning, and electrophysiology measurements) by utilizing DMD simulations. Upon developing the improved model of the OWF state, we test the validity the new OWF structure by carrying out cysteine cross-linking experiments on numerous residue pairs predicted to line the pore. Single channel recordings are performed to determine the functional impact of selected pore-lining residue pairs upon cross-linking. The IWF state of CFTR is constructed by using I-TASSER modeling [21,22] based on the template structure of Thermotoga maritime TM287/288 [14]. Note that, TM287/288, like CFTR, contains asymmetric NBDs, with one degenerate and one consensus nucleotide-binding site. While we initially select the TM287/288 structure as template for reconstructing the IWF conformation, I-TASSER utilizes multiple other ABC transporter structures to develop the IWF model of CFTR (S15 Fig). Upon the refinement of the OWF model and the derivation of the IWF model based on homology modeling, to produce physiologically relevant, optimized structures of CFTR in both conformations, we carry out a step-by-step MD simulation procedure. The final models provided here are obtained from ~200 ns MD equilibration. This precaution is taken to assure that any bias (that may have been introduced from experimental restraints) during the fine-tuning step and/or homology modeling be eliminated. Detailed procedures followed for developing the OWF and IWF models is described in the supplemental information (SI) (S1 Text). In-depth experimental designs relating to cysteine cross-linking and single channel recording experiments are provided in SI. The coordinates of the OWF and IWF models (at the end of 200 ns MD simulations) can be downloaded from the following link: http://troll.med.unc.edu/cftr/.

## Supporting information

S1 Text Materials and methods. (DOCX)

S1 Table Pore lining residues (obtained from previous biochemical and biophysical experiments) for fine-tuning our earlier OWF model. (DOCX)

S2 Table Predicted residue pairs for model validation using cysteine cross-linking experiments. (DOCX)

S3 Table List of control residue pairs for testing via cysteine cross-linking experiments. (DOCX)

S4 Table Comparison between IWF homology model versus cryo-EM structure of CFTR (PDBID: 5UAK) with tested residue pairs using cysteine cross-linking experiments. (DOCX)

S5 Table Distance comparison between the IWF homology model, MD-derived structure and cryo-EM structure within the NBD regions versus published cysteine cross-linking results. (DOCX)

S1 Fig Pore lining residues residing on transmembrane helices 6, 9 and 12. A. outward- and B. inward-facing conformations. The helices are shown with cartoon: Cyan–TMH6, Green–TMH9 and Orange–TMH12. The individual residues are labeled and shown in stick representation.
(TIF)

S2 Fig Root mean squared deviation (RMSD) of protein as a function of simulation time. Black curve depicts the RMSD of outward-facing state, and maroon curve shows the RMSD of the inward-facing state with respect to their initial computationally modeled structures. The inset represents the RMSD of protein in OWF (black) and IWF (maroon) states with respect to their final structures obtained at the end of 200 ns simulations. The overall structures do not alter significantly during the last 100 ns production runs as depicted by the RMSD change of ~ 2 Å in both cases. Therefore, the equilibrated structural modes of OWF and IWF configurations can be represented by the 200^th^ ns ones. The representative structures of the OWF conformations from last 100 ns MD runs are depicted in S13 Fig.
(TIF)

S3 Fig Root mean squared deviation (RMSD) of different domains of CFTR as a function of simulation time. The RMSD of the OWF (upper) and IWF (lower) conformations are evaluated using the entire 200 ns MD trajectory in comparison with the initially computationally built structures before they are subjected to equilibrations. The higher RMSD values in the RMSD of the full-length proteins arise from the disordered regions of NBD1 (OWF state) and NBD2 (IWF state).
(TIF)

S4 Fig Western blots of cysteine cross-links of additional residue pairs in the putative CFTR pore. Among the tested 29 pairs, 23 pairs show cross-links upon treatment with MTS reagents of various lengths. Shown here are 7 pairs of cross-links. Immature core-glycosylated CFTR is marked as band b; mature complex-glycosylated CFTR as band c; band x represents cross-linked mature protein. Overall correspondence between the relative distances between all the residue pairs retrieved from cross-linking experiments and the newly refined outward facing CFTR structure is 100% (refer to S2 Table). Note that, the putative CFTR pore spans the entire pore region including the extracellular, membraneous and intracellular regions.
(TIF)

S5 Fig Cross-linking of cysteine pairs in the putative CFTR pore in inward facing state. Left: refined inward facing state of CFTR model exhibiting examples of residue pairs XL1, XL2, XL3, XL4 and XL5 (spheres) used for testing the reliability of the CFTR pore. The inward facing CFTR structure presented here is obtained from 200 ns MD simulation. Right: the average distances between each residue pairs. This figure is related to Fig 3 and the corresponding western blots are exhibited in the same.
(TIF)

S6 Fig Blockage of currents of single cysteine mutant channels by bifunctional MTS reagents and of the R352C/D993C cysteine pair variant by monofunctional MTSBn. (A) Blockage of W1145C channel by 20 μM M5M. Three Cys-less W1145C channel recordings with the wild type CFTR ion pore conductance of 12.3 pS is shown. The technical gap in the recording is shown as a bar and represents a 2 minutes interval needed for 20 μM M5M MTS reagent application to the cis side of the bilayer. All three channels were converted to a nonconductive state shortly after recording resumed. (B) Blockage of D993C channel by 20 μM M5M. Two channel recordings of Cys-less D993C construct with the pore conductance of 14.5 pS. The negative charge removal increases pore conductance in comparison with the wild type CFTR only slightly. The gap in the recording is shown as a bar and represents about 2 minutes time interval needed for the 20 μM M3M application at the cis side. Both channels were turned into the nonconductive state shortly after recording resumed. (C) Blockage of R352C/D993C channel by 50 μM MTSBn. Vertical scale bar of 1pA and horizontal scale bar of 10s are common for all traces. The chemical structures of M5M, M3M and MTSBn reagents are shown on the left side of each tracing.
(TIF)

S7 Fig R347C cross-links with S1141C and D993C. **(**A) Relative positions of R347 in TMH6, D993 in TMH9, S1141 in TMH12 in outward facing state. (B) Western blot of untreated Cys-less CFTR R347C/S1141C constructs (lane 1) and treated with 20 μM of MTS cross linkers of different length (lanes 2–4). No change in mature band position of untreated sample (lane 1) suggests that no tight contact exists between cysteines at these positions. Thus, the inter-residue distance fluctuates significantly. (C) 10 minute single channel recording of Cys-less R347C/S1141C with γ = 4.1 pS under native conditions (reduction by 5 mM DTT) without any treatments with other thiol reagents. No opening is observed during the recording period after treatment with 20 μM M5M MTS reagent (lower tracing). **(**D) Relative positions of R347 in TMH6, D993 in TMH9, S1141 in TMH12 in inward facing state. (E) Western Blot of untreated Cys-less CFTR R347C/D993C constructs (lane 1) and treated by 20 μM of MTS cross linkers of different lengths (lanes 2, 3, and 4). No change in mature band position without treatment (lane 1) indicates that there is no spontaneous S-S bond formation between R347C and D993C while only M17M causes a strong shift of mature c-band to the x-band. (F) Single channel recordings of R347C/D993C with γ = 4.1 pS under the reducing conditions induced by 5 mM DTT. Channel conductance is lost as a result of cross-linking by 20 μM M17M (lower tracing).
(TIF)

S8 Fig Cross-linking of W356C/L989C and W356C/S1149C residue pairs. (A) Locations of W356, L989 and S1149 residues residing in TMHs 3, 9 and 12, respectively in A) outward facing state. (B) Western blot of Cys-less CFTR W356C/L989C before (lane 1) and after treatment with MTS (20 μM) reagents (lanes 2–6). No change in c band position without treatment suggests that no tight contact exists between cysteines at these positions. Strong shift of c to x band position is observed after treatment by cross linkers M2M through M17M. (C) Recordings of two independent Cys-less W356C/L989C CFTR ion channels with γ = 12.8 pS are shown on left: these independent channels are labeled as I, and II; channel closing is labeled with C. Recording of the same membrane after adding 20 μM M5M cross-linker at the “cis” side is shown on right. A 20s interruption in the recording used for cross-linker application and stirring is shown by the dotted line. (D) Positions of W356, L989 and S1149 in the inward facing state. E) Western blot of Cys-less CFTR W356C/S1149C before (lane 1) and after treatment with 20 μM MTS cross-linkers (lanes 2–6). (F) Recording of five independent Cys-less W356C/S1149C CFTR channels with γ = 12.9 pS (shown on left): the five channels are labeled as I, II, III, IV and V; channel closing is indicated by C. Recording of the same multi-channel membrane after application of 20 μM M3M at the “cis” side is on the right. There is a 20s interruption in the recording for cross-linker application, the dashed line shows stirring. Vertical (X) and horizontal (Y) axes scale bars represent 10s and 1 pA, respectively.
(TIF)

S9 Fig Structural representation of the proposed *selectivity filter*. (A) outward- and (B) inward-facing conformations. The TMHs 1 (pink), 6 (cyan), and 12 (orange) are color-coded. These structures are the final conformations of the CFTR channels after 200 ns molecular dynamics simulations.
(TIF)

S10 Fig Alignment of MSD and NBD regions of our CFTR channel (in IWF state) versus the cryo-EM structure (PDBID: 5UAK). A. homology model based CFTR structure versus 5UAK, and B. channel structure obtained after 200 ns MD equilibration versus 5UAK. The magenta colored structure represents cryo-EM model and our MSD1, MSD2, NBD1 and NBD2 structures are depicted with cyan, grey-white, green and dark gray colors.
(TIF)

S11 Fig Alignment of all TM helices from our CFTR channels (in IWF state) versus the cryo-EM structure (PDBID: 5UAK). A. The homology model based structure versus 5UAK and B. CFTR structure at the end of 200 ns MD equilibration versus 5UAK. The magenta colored helices represent cryo-EM model and our TMHs are depicted with following colors: TMH1 –wheat, TMH2 –green, TMH3 –olive, TMH4 –yellow, TMH5 –salmon, TMH6 –sea green, TMH7 –raspberry, TMH8 –pink, TMH9 –cyan, TMH10 –blue, TMH11 –purple, TMH12—orange.
(TIF)

S12 Fig Compatibility of CFTR structure pore with functional data (IWF state) and cryo-EM model of zebrafish CFTR (PDBID: 5TSI). Residues accessible from the intracellular side of the cell: S307, F311, V345, M348, A349, R352, Q353, T1112, S1141, T1142, Q1144, W1145, V1147, N1148, S1149 (Cβ atoms are presented using blue spheres); residues accessible from the extracellular side of the cell: R104, L323, A326, R334, K335, I336, I1121, T1122, I1131, and I1132 (Cβ atoms are presented using magenta spheres); and the pore lining residues: Q98, P99, L102, F337, T338, S341, I344, T1115, S1118, N1138, and M1140 (Cβ atoms are presented using green spheres).
(TIF)

S13 Fig The relative Cβ distances between residue pairs residing within the NBD interface previously tested by cross-linking (55). A. OWF structure (upper panel) and B. IWF structure. The average distances and the standard deviations are quantified by sampling 200 ns MD trajectory.
(TIF)

S14 Fig Representative CFTR structures (in OWF state) from molecular dynamics based on RMSD based clustering. Representative centroid structure of (A) cluster 5 (green) versus 1 (pink) (B) cluster 5 (green) versus 2 (orange) (C) cluster 5 (green) versus 3 (ice blue) and (D) cluster 5 (green) versus 4 (white). The representative structures from five different clusters are very similar suggesting that the transition between different conductive and non-conductive states is not attained during the span (~ 200 ns) of the simulation. Longer simulations are required for studying channel gating and associated alternate conformations of the channel. In both cases, the clustering is obtained by isolating structures within a RMSD of ~ 2 Å of one another. Note that only backbone atoms are considered. The representative structure presented here is simply the centroid of each cluster. Cluster 5 is taken as the reference model as it has the highest population. Clustering analyses yields five clusters from the entire production run based on our clustering criterion.
(TIF)

S15 Fig Protein templates used for reconstructing IWF state of CFTR by I-TASSER. The sequence alignment of CFTR versus the structural templates applied in model building is presented.
(TIF)
