# Supplementary material for: Transmembrane helical interactions in the CFTR channel pore
Source: PLoS Comput Biol. 2017 Jun 22;13(6):e1005594. doi: 10.1371/journal.pcbi.1005594 (PMC5501672; doi:10.1371/journal.pcbi.1005594)
Supplement: S1 Text — (DOCX) [file pcbi.1005594.s001.docx]

**Supplemental Information**

**Transmembrane helical interactions in the CFTR channel pore**

Jhuma Das^1^, Andrei A. Aleksandrov^1,2^, Liying Cui^1,2^, Lihua He^1,2^, John R. Riordan^1,2,#^, Nikolay V. Dokholyan^1,2, #^

**1** Department of Biochemistry and Biophysics, University of North Carolina at Chapel Hill, Chapel Hill, North Carolina, United States of America, **2** Cystic Fibrosis Treatment and Research Center, University of North Carolina at Chapel Hill, Chapel Hill, North Carolina, United States of America.

^#^To whom correspondence should be addressed: John R. Riordan ([john_riordan@med.unc.edu](mailto:john_riordan@med.unc.edu)) and Nikolay V. Dokholyan ([dokh@unc.edu](mailto:dokh@unc.edu))

**S1 Text. Materials and Methods**

**Modeling inward-facing state of CFTR**

Homology model of inward facing (IWF) CFTR is generated using the I-TASSER server (1,2) based on the template structure, TM287/TM288 (PDB ID: 3QF4 and resolution ~ 2.9 Å) (3). The sequence similarity between CFTR and the ABC transporter TM287/TM288 is ~ 20%. I-TASSER (ranked the best server for protein structure prediction in the latest Critical Assessment of Techniques for Protein Structure Prediction (CASP7) uses a combined approach of threading, *ab initio* folding, and structure refinement for constructing reliable homology based models. The strength of I-TASSER the software lies in generating full- length model of proteins by excising continuous fragments from threading alignments and then reassembling them using replica-exchanged Monte Carlo simulations. On top of the template 3QF4 provided by us, I-TASSER incorporated several other templates including the structures of ABC transporter MsbA (PDBID: 3B60), and P-glycoprotein (PDBID: 3G61). The sequence alignment of all the templates used to generate the IWF state of CFTR is illustrated in S14 Figure. From the five models generated by I-TASSER, we choose the model with the highest C-Score (= -1.70) and the highest TM-Score (=0.51±0.14) for our study. The detailed definitions of C- and TM-scores are vital to determining the reliability of the new structure. Briefly, C-score provides the confidence score for estimating the quality of predicted models by I-TASSER and is estimated via the significance of threading template alignments and the convergence parameters of the structure assembly simulations. Typical range of C-score is between -5, and 2: lower C-score implies the model with a high confidence and vice-versa. A TM-score > 0.5 indicates a model of correct topology, while TM-score < 0.17 means a random similarity. The C- and TM-scores reported by I-TASSER for the closed state of CFTR shows good confidence in the model prediction. Our final IWF CFTR structure excludes R-domain since there is no structural information available to accurately build this disordered domain. It should be noted that, unlike the outward facing state of CFTR, the functional data concerning to CFTR closed state structure is scarce. Therefore, refining the closed state based on experimental information is beyond the scope of this study. Further modification will be pursued to design an improved closed model of CFTR utilizing the integrated experimental-computational approach described below when we acquire more structural data from experiments.

**Refinement of putative pore of CFTR using functional data**

Refinement of the putative pore region of the of the outward facing state of CFTR is achieved by incorporating functional data available in the literature as well as the electrophysiological experiments (4) performed in Riordan lab (S1 Table). We visually inspect our previously constructed OWF CFTR structural model (5) with Pymol (The PyMOL Molecular Graphics System, Version 1.5.0.4 Schrödinger, LLC) to identify residues that do not agree with experimentally determined residue orientation in the context of the pore interior. Using the tools in Pymol, we reorient the helix associated with the mismatched residue to correct its orientation. Next, we reconstruct new peptide bonds between the adjusted residue (or residues) with the adjacent residues by employing DMD simulations (6,7). In each simulation, static constraints are applied to the entire protein except for the modified region of the protein that is involved in peptide bond formation to prevent undesirable structural rearrangement in the whole protein while the unconstrained part is optimized to obtain an energy minimized structure. Upon implementation of each constraint, the systems are subjected to short-time equilibration to resolve any unphysical perturbation/distortion that might result due to addition of those restraints. This procedure is repeated until the structure is consistent with all available functional data. After including all modifications via the steps described above, we run the refined model through our in-house developed software Chiron (8) to obtain a clash-free, minimized model structure. We evaluate the quality of the final structure by means of another in-house developed tool Gaia (9) against high-resolution crystal structures. The Gaia score for our newly built structures is well within the expected values for all the parameters derived from high-resolution crystal structures of proteins. For completion purpose, we have also reconstructed the N- and C-termini using homology modeling technique (I-TASSER) and DMD simulations is utilized to reconnect these segments to the new OWF model. However, to test the validity of these segments, new experiments are warranted. Our current work is focused on reconstructing the pore region. The predictability associated with N-, and C-terminal models and the related methodology will be published elsewhere upon detailed experimental verification.

**Molecular dynamics simulation protocol**

Upon modeling the atomistic wild type OWF and IWF CFTR structures, we perform all atom molecular dynamics (MD) simulations using the GROMACS package, version 4.6.3 (10-12). Using the Membrane Builder plugin of CHARMM-GUI web server (13), we generate a pre-equilibrated POPE/POPS lipid bilayer (with POPE:POPS at a 2:1 ratio following single channel measurement criteria). We solvate the protein with pre-equilibrated POPE/POPS lipid bilayer using the g_membed program in GROMACS package. This program ascertains that all water and lipid molecules that cause steric clashes with the protein are removed after insertion of the protein into the hydrated membrane. The correct alignment and the orientation of the protein are achieved by applying the results obtained through the PPM (Positioning of Proteins in Membranes) method offered with the OPM (Orientations of Proteins in Membranes) database (14). PPM server ensures optimal protein alignment in the membrane by accounting for the hydrophobic, hydrogen bond and electrostatic interactions of proteins with the water-membrane environment. Next, the entire system is solvated with TIP3P water molecules using the GROMACS *genbox* program. To maintain 0.3 M salt concentrations (same salt concentration is used in our experiments), and to neutralize the system, we add sodium (Na^+^) and chloride (Cl^-^) ions to the system using the *genion* program available in GROMACS. Our final OWF model system consists of one wild type CFTR molecule, 469 POPE and 234 POPS lipid molecules, 82897 water molecules, 869 Na^+^ ions, and 618 Cl^-^ ions. The size of the model system is 14.1 x 15.3 x 16.2 nm^3^. Our final IWF model system consists of one wild type CFTR molecule, 581 POPE and 289 POPS lipid molecules, 98298 water molecules, 1080 Na^+^ ions, and 814 Cl^-^ ions. The size of the model system is 15.8 x 15.1 x 17.2 nm^3^.

We perform all simulations at constant pressure (p=1atm) and temperature (T=310K) with the CHARMM-36 force field (15) for proteins with additional parameters for lipids (13). We use weak Nosé-Hoover temperature coupling (16,17) to maintain a temperature of 310 K with coupling time constant 0.5 ps. We use semi-isotropic Parinello-Rahman pressure coupling (18,19) to maintain a pressure of 1 bar, with a relaxation constant of 5 ps. The time step of each simulation is 2 fs. We apply a cut-off of 1.2 nm for van der Waals interactions. We use the Particle Mesh Ewald sum(20) for long-range electrostatic interaction with a cut-off distance of 1.2 nm and a grid spacing of 1.6 nm. We use the LINCS algorithm to constrain bond lengths. We apply periodic boundary conditions to eliminate the finite size effect. We carry out 20,000 minimization steps for each system with a steepest descent algorithm. Following the minimization, we perform 100 ns of MD simulation to ensure system equilibration for each simulation run with two models. Then 100 ns of production simulations are executed for each system. The average root mean squared deviations of the protein backbone atoms (excluding R domain and the C- and N- termini) approach steady-state values (~ 8.3 Å and 7.6 Å for OWF and IWF systems, respectively) within 100 ns (S2 Figure), and remain stable during the 100 ns production runs (inset of S2 Figure) indicating that the system is equilibrated after 100 ns. The final equilibrated structures of the OWF and IWF CFTR are presented in Fig. 1. Note that, CFTR is a unique multi-domain protein consisting of partially structured MSDs, NBDs, several connecting loops, a disordered R-domain, and many other disordered segments. It is impossible to predict the structure of the disordered regions without a template with significantly higher sequence similarity. Therefore, it is not surprising that the protein exhibits higher RMSD. The high RMSD (~ 8 Å) is mainly associated with the disordered regions in the NBD regions as well as the extra- and intra-cellular loops and other structurally disordered regions in both cases. By computing RMSDs between different domains (e.g., MSDs, and NBDs), we observe that the MSDs do not undergo major rearrangements (< 5 Å) during the 200 ns simulations in both conformations (S3 Figure). On the other hand, the initial increase in the RMSDs for the NBD1 region in OWF and for NBD2 in IWF structures are due to their relative translational motion (in longitudinal and lateral directions) with respect to their initial positions. Furthermore, while partially structured, NBD1 and NBD2 consist of various unstructured regions (e.g., regulatory insertion loop, structurally diverse region in NBD1, etc.) that contribute to corresponding higher RMSDs. For instance, excluding the regulatory insertion loop (RI) from NBD1 (in the OWF conformation) reduces the RMSD of the same by ~ 3 Å in turn dropping the overall RMSD of the same to ~ 5 Å. Note that, after the first 100ns, the overall RMSD reaches a steady average value for both conformations indicating that the corresponding structures attains equilibrium. It should also be noted that, the relative positions of the NBD regions as well as their locations with respect to the intracellular loops derived from our MD-generated structure align well with the cryo-EM structure (21) (S5 Table). The asymmetric opening at the NBD interface is also preserved in our models as seen in the cryo-EM structure (S5 Table). MD simulations are conducted precisely as a means to obtain lowest energy structures by eradicating any bias and/or distortions that might have been introduced to the systems’ molecular structures upon implementation of the experimental constraints.

**Testing predictability of CFTR pore structures through cysteine cross-linking**

To validate the predictability of newly built outward facing CFTR structure, we select numerous residue pairs spanning the ion conduction pathway and verify the inter-residue distances between each pair using cysteine cross-linking experiments. All the cross-linker distances obtained from experiments are compared versus the inter-residue distances derived from the models (S2-S3 Tables). A few cysteine pairs are used as “control” pairs (S3 Table). According to the models, we should not be able to cross-link these “control” pairs. These set of experiments are done to demonstrate the specificity of cross-linking techniques. For these residue pairs, either one of the two partnering residues is turning away from channel pore or are not aligned correctly with their partner residue, thus making them inaccessible to cross-linking or their relative distance is larger than ~ 25.0 Å (outside the distance covered by the largest cross-linker M17M used in our study). Cross-linking distances obtained from 23 cysteine-substituted residue pairs correspond very well with the model-derived distances (S2 Table), thus validating the credibility of our CFTR pore structures. The six cysteine-substituted pairs used as controls (S3 Table) also match model predictions. Note that, our cysteine cross-linking studies utilize Cys-less CFTR constructs instead of the wild type protein to guarantee that the cross-linking occurs only at the targeted residue pair positions. This precaution is taken to ensure that the measurements obtained in our study are not contaminated by other undesirable cross-links or chemical modification elsewhere in the protein. It is safe to use Cys-less CFTR constructs for our purposes since we previously showed that our Cys-less constructs have similar functional characteristics as wild type channels in physiological conditions (22).

**Western blot**

Western blot of Cys-less CFTR constructs with specific residue pairs are obtained by following the steps outlined below.

Construction and expression of mutants. cDNA coding Cys-less CFTR (5,23) is produced in the pcDNA3 cloning vector by using the Stratagene QuikChange protocol. All of the cysteine pairs are introduced in this Cys-less construct with the same Stratagene QuikChange method. For mammalian cell expression, the cysteine pairs are transiently transfected into HEK 293 cells using jetPEI (Polyplus) transfection reagent according to the manufacturer's protocol. To increase CFTR maturation, VX809 (3μM) is added to the media and cells are moved to 27°C for 24 hours after transfection. Cells are harvested 48 hours after transfection for cross-linking experiments or membrane preparation.

Isolation of membrane vesicles. Membrane vesicles are isolated from HEK293 cells expressing variants of CFTR. Cell pellets are re-suspended in ice-cold hypotonic lysis buffer [10 mM Hepes (pH 7.2), 1 mM EDTA, 2 µg/ml leupeptin, 4 µg/ml aprotinin, 250 µg/ml benzamidine, 100 µg/ml Pefabloc, 7 µg/ml E64]. After incubation on ice for 15 min, cells are lysed by 7 strokes in a Dounce homogenizer, and an equal volume of sucrose buffer [500 mM sucrose, 10 mM Hepes (pH 7.2)] is added followed by a further 7 strokes. To pellet microsomes, the postmitochondrial supernatant (10,000×g, 10 min) is centrifuged at 100,000 × g for 45 min. The sedimented microsomes are re-suspended in a buffer [250 mM sucrose, 10 mM Hepes, 5 mM MgCl_2_ (pH 7.2)] to yield a total protein concentration of 1-3 mg/ml. Vesicles with a uniform diameter of ~1 µm are obtained after sonication (3 × 20 s) and stored at −80°C until used.

Phosphorylation of CFTR. To stimulate phosphorylation of CFTR in cells, cells are incubated for 15 min in PBS supplemented with 10 µM forskolin, 100 µM DiBu-cAMP, and 1 mM 3-isobutyl-1-methylxanthine at 37°C. This stimulation mixture is also present during the cross-linking reaction. To phosphorylate CFTR in membrane vesicles, membranes are treated with 100 units/ml PKA (Promega) in the presence of 2 mM ATP for 15 min at room temperature. The membranes are then sonicated and incubated at room temperature for another 15 minutes to ensure complete phosphorylation.

Cross-linking and Western blotting. CFTR-expressing cells grown on 35-mm tissue culture dishes are harvested, washed twice in PBS, and re-suspended in 80 µl of PBS. Ten microliters of cell suspension is mixed with 20 µl of PBS or PBS containing 300 µM cross-linkers to yield a final concentration of 200 µM. Bifunctional cross-linkers from Toronto Research Chemicals are used: M1M (1,1-methanediyl bismethanethiosulfonate), M3M (1,3-propanediyl bismethanethiosulfonate), M5M (1,5- butanediyl bismethanethiosulfonate), M8M (1,8-pentanediyl bismethanethiosulfonate), and M17M (3,6,9,12,15-pentaoxaheptadecane-1, 17-diyl bismethanethiosulfonate). To cross-link CFTR in membrane vesicles, the same procedure is employed. The cross-linking reaction is terminated by addition of 4μl of 10X with Laemmli sample buffer with or without DTT. The samples are resolved by SDS/PAGE, and Western blots probed with anti-CFTR mAb596. CFTR is detected with secondary antibodies labeled with infrared dyes by using the Odyssey infrared scanner (Licor, Inc.). Note that, for each cross-linking pair, we perform the experiments two or three times to examine their consistency and the results are generally reproducible.

**Single channel recording**

Single channel experiments are performed as described previously^11^. Ion channels are transferred into the preformed lipid bilayer by spontaneous fusion of membrane vesicles with preformed planar lipid bilayer consists of 1-palmitoyl-2-oleoyl-sn-glycero-3-phospho-ethanolamine and 1-palmitoyl-2-oleoyl-sn-glycero-3-phosphoserine (Avanti Polar Lipids) at a 2:1 ratio, which is painted over a 0.2-mm aperture in a Teflon partition between cis and trans compartments of a chamber. The membrane vesicles containing CFTR variants are phosphorylated for 20 min at +4°C by 100 U ml^−1^ of PKA and 2 mM ATP in buffer containing 250 mM sucrose, 5 mM MgCl_2_, 10 mM Hepes, pH 7.3. To maintain uniform orientation of the functional ion channels in the lipid bilayer, 10 μl membrane vesicles (1 mg ml^−1^ total protein in the vesicles stock solution), 2 mM ATP and 100 Uml^−1^ of PKA catalytic subunits are added to the ‘cis’ compartment only.

Single channel currents are measured at −75 mV under voltage-clamp conditions using an Axopatch 200B amplifier (Axon Instruments/Molecular Devices, Sunnyvale, CA, USA), where the membrane voltage potential of −75 mV is the difference between cis and trans (ground) compartments. All measurements are done at 30°C in symmetrical salt solution containing: 300mM Tris/HCl pH 7.2, 3mM MgCl2 and 1mM EGTA. Heating and temperature control are established using a Temperature Control System TC2BIP (Cell Micro Controls, Norfolk, VA, USA).

The output signal is filtered with an 8-pole Bessel low-pass filter LPBF-48DG (NPI Electronic, Tamm, Germany) with a cut-off frequency of 50 Hz, digitized with a sampling rate of 500 Hz and recorded with pClamp 9.2 software (Axon Instruments). Origin 7.5 software (Origin Lab Corp., Northampton, MA, USA) is used to fit all-points histograms by multi-peak Gaussians. We define single-channel current as the distance between peaks on the fitting curve and is used for determining the single-channel conductance (γ). The probability of the single channel being open (Po) is calculated as a ratio of the area under the peak for the open state to the total area under both peaks on the fitting curve.

The pairs of cysteine residues are introduced into Cys-less CFTR construct to investigate possible intra-molecular contacts appeared in CFTR functional cycle. Cross-link between cysteine pairs are monitored in real time as a functional change in the CFTR single channel current induced by bifunctional methane-thiosulfonate (MTS reagents, Toronto Research Chemicals) reagents with spacer arms ranging from 3.9 to 24.7 Å. The ability of cross-link between cysteine pair to occur is considered as an evidence of the close proximity of the residue pairs in question. Note that different concentrations of cross linkers are used for electrophysiology and Western Blot experiments. The low 20 - 50 μM concentration is used for electrophysiology to obtain results within 2-5 minutes while avoiding side effects (24) such as non-specific interactions with other residues. The high 200 μM is used for the Western Blots to maintain uniform concentrations for the cross-linkers of different lengths and efficiency.

**Construction of pore radius profile**

We sample 250 structures at even intervals from each simulation trajectories (100 ns production run) described above. For each structure, we estimate the radius along the axis of the channel pore using HOLE (25) for both OWF and IWF conformations. We average these results to account for the stochastic effects of sampling, and obtain a final profile reflecting the shape of the pore of both the IWF and OWF CFTR systems.

**References**

1. Zhang Y. I-TASSER server for protein 3D structure prediction. BMC Bioinformatics. 2008; 9(1):40.

2. Roy A, Kucukural A, Zhang Y. I-TASSER: a unified platform for automated protein structure and function prediction. Nat Protoc. 2010; 5(4):725–38.

3. Hohl M, Briand C, Grütter MG, Seeger MA. Crystal structure of a heterodimeric ABC transporter in its inward-facing conformation. Nature Publishing Group. Nature Publishing Group; 2012; 19(4):395–402.

4. Linsdell P. Functional architecture of the CFTR chloride channel. Mol Membr Biol. 2014; 31(1):1–16.

5. Serohijos AWR, Hegedus T, Aleksandrov AA, He L, Cui L, Dokholyan NV, et al. Phenylalanine-508 mediates a cytoplasmic-membrane domain contact in the CFTR 3D structure crucial to assembly and channel function. Proceedings of the National Academy of Sciences. 2008; 105(9):3256–61.

6. Dokholyan NV. Discrete molecular dynamics studies of the folding of a protein-like model. Folding & Design. 1998; 3:577–87.

7. Ding F, Tsao D, Nie H, Dokholyan NV. Ab Initio Folding of Proteins with All-Atom Discrete Molecular Dynamics. Structure. 2008; 16(7):1010–8.

8. Ramachandran S, Kota P, Ding F, Dokholyan NV. Automated minimization of steric clashes in protein structures. Proteins. 2010;79(1):261–70.

9. Kota P, Ding F, Ramachandran S, Dokholyan NV. Gaia: automated quality assessment of protein structure models. Bioinformatics. 2011; 27(16):2209–15.

10. Lindahl E, hess B, van der Spoel D. GROMACS 3.0: a package for molecular simulation and trajectory analysis. J Mol Model. 2001; 7:306–17.

11. van der Spoel D, Lindahl E, hess B, Groenhof G, Marsden BD, Berendsen HJC. GROMACS: Fast, flexible, and free. J Comput Chem. 2005; 26(16):1701–18.

12. hess B, Kutzner C, van der Spoel D, Lindahl E. GROMACS 4:  Algorithms for Highly Efficient, Load-Balanced, and Scalable Molecular Simulation. J Chem Theory Comput. 2008; 4(3):435–47.

13. Jo S, Kim T, Iyer VG, Im W. CHARMM-GUI: A web-based graphical user interface for CHARMM. J Comput Chem. 2008; 29(11):1859–65.

14. Lomize MA, Pogozheva ID, Joo H, Mosberg HI, Lomize AL. OPM database and PPM web server: resources for positioning of proteins in membranes. Nucleic Acids Research. 2011; 40(D1):D370–6.

15. Huang J, MacKerell AD Jr. CHARMM36 all-atom additive protein force field: Validation based on comparison to NMR data. J Comput Chem. 2013; 34(25):2135–45.

16. Nosé S. A unified formulation of the constant temperature molecular dynamics methods. J Chem Phys. 1984; 81(1):511.

17. Hoover WG. Hoover_coupling_PhysRevA.31.1695. Physical Review A. 1985; 31(3):1695–7.

18. Parrinello M, Rahman A. Parinello-Rahman_PhysRevLett.45.1196. 1980; 45(14):1196–9.

19. Parrinello M. Polymorphic transitions in single crystals: A new molecular dynamics method. J Appl Phys. 1981; 52(12):7182.

20. Essmann U, Perera L, Berkowitz ML, Darden T, Lee H, Pedersen LG. A smooth particle mesh Ewald method. J Chem Phys. 1995; 103(19):8577.

21. Zhang Z, Chen J. Atomic Structure of the Cystic Fibrosis Transmembrane Conductance Regulator. Cell. 2016; 167(6):1586-97.

22. Cui L, Aleksandrov L, hou Y-X, Gentzsch M, Chen J-H, Riordan JR, et al. The role of cystic fibrosis transmembrane conductance regulator phenylalanine 508 side chain in ion channel gating. The Journal of Physiology. 2006; 572(2):347–58.

23. He L, Aleksandrov AA, Serohijos AWR, Hegedus T, Aleksandrov LA, Cui L, et al. Multiple Membrane-Cytoplasmic Domain Contacts in the Cystic Fibrosis Transmembrane Conductance Regulator (CFTR) Mediate Regulation of Channel Gating. Journal of Biological Chemistry. 2008; 283(39):26383–90.

24. Li M-S, Demsey A, Qi J, Linsdell P. Cysteine-independent inhibition of the CFTR chloride channel by the cysteine-reactive reagent sodium (2-sulphonatoethyl) methanethiosulphonate. British Journal of Pharmacology. 2009; 157(6):1065–71.

25. Smart OS, Goodfellow JM, Wallace BA. The Pore Dimensions of Gramicidin A. 1993; 65:2455–60.
