# Supplementary material for: Transmembrane helical interactions in the CFTR channel pore
Source: PLoS Comput Biol. 2017 Jun 22;13(6):e1005594. doi: 10.1371/journal.pcbi.1005594 (PMC5501672; doi:10.1371/journal.pcbi.1005594)
Supplement: S1 Table — (DOCX) [file pcbi.1005594.s002.docx]

**S1 Table. Pore lining residues (obtained from previous biochemical and biophysical experiments) for fine-tuning our earlier OWF model**(5)**.**

| TMH | 3 | 6 | 9 | 12 |
| --- | --- | --- | --- | --- |
| Residue positions | K190  F191  D192  E193  G194  A196  L197  F200  G213  I215  L218 | I331  L333  R334  K335  I336  F337  T338  S341  I344  V345  M348  A349  R352  Q353  W356  T360  D363 | L986  P988  L989  T990  D993  L997  I1000  G1003  V1008  A1009  V1010  Q1012 | G1127  V1129  I1131  I1132  N1138  M1140  S1141  T1142  Q1144  W1145  V1147  N1148  S1149  D1152  D1154  L1156 |
