# Supplementary material for: Transmembrane helical interactions in the CFTR channel pore
Source: PLoS Comput Biol. 2017 Jun 22;13(6):e1005594. doi: 10.1371/journal.pcbi.1005594 (PMC5501672; doi:10.1371/journal.pcbi.1005594)
Supplement: S2 Table — (DOCX) [file pcbi.1005594.s003.docx]

**S2 Table. Predicted residue pairs for model validation using cysteine cross-linking experiments.**

| Predicted pairs for cross-linking | Cβ-Cβ distance in outward facing model (Å) | | Cβ-Cβ distance in inward facing model (Å) | | Shortest MTS cross-linker for full conversion of c 🡪 x band (Å) |
| --- | --- | --- | --- | --- | --- |
|  | Static | MD | Static | MD |  |
|  | Static | MD | Static | MD |  |
| W356C/D1152C | 14.6 | 13.5±0.43 | 17.1 | 18.9±1.11 | M17M |
| W356C/S1149C | 14.9 | 14.2±0.50 | 16.9 | 15.2±0.64 | M8M |
| W356C/W1145C | 12.7 | 12.1±0.40 | 18.1 | 10.1±1.08 | M17M |
| R352C/D1152C | 14.9 | 11.9±0.66 | 20.3 | 19.9±0.98 | M17M |
| R352C/W1145C | 9.0 | 6.3±0.37 | 9.5 | 6.1±1.08 | M3M |
| R347C/S1141C | 14.5 | 9.6±0.39 | 20.0 | 8.80±0.27 | M5M |
| S341C/T1134C | 12.3 | 5.1±0.30 | 15.9 | 5.6±0.53 | M3M |
| T338C/I1131C | 18.8 | 12.2±0.42 | 14.5 | 9.4±0.60 | M17M |
| T338C/G1127C | 17.8 | 9.8±0.42 | 15.1 | 11.6±1.03 | M17M |
| F337C/T1134C | 12.9 | 10.0±0.32 | 18.1 | 11.2±0.49 | M17M |
| F337C/I1131C | 14.2 | 9.6±0.40 | 18.9 | 12.9±0.68 | M3M |
| F337C/G1127C | 13.2 | 6.6±0.36 | 19.0 | 15.5±1.22 | M1M |
| R334C/G1127C | 14.3 | 8.1±0.52 | 14.8 | 14.9±0.88 | M1M |
| R347C/D993C^#^ | 10.6 | 14.3±0.52 | 33.0 | 29.6±0.53 | M1M |
| R352C/Q996C | 12.7 | 10.4±0.49 | 23.4 | 18.6±0.62 | M8M |
| R352C/D993C^*^ | 9.0 | 9.4±0.43 | 26.4 | 23.5±0.73 | M5M |
| W356C/L989C | 13.0 | 16.1±0.64 | 23.5 | 22.7±0.63 | M2M |
| R251C/D1152C | 20.9 | 19.7±0.78 | 24.1 | 24.2±1.17 | M17M |
| K190C/L1156C | 18.2 | 19.4±0.59 | 24.0 | 23.7±0.62 | M8M |
| E193C/L989C | 14.9 | 18.8±0.84 | 29.1 | 21.2±0.43 | M17M |
| E193C/L986C | 20.0 | 25.8±0.90 | 28.5 | 25.6±0.52 | M8M |
| K190C/K978C | 25.0 | 31.9±0.88 | 24.4 | 18.7±0.50 | M8M |
| N186C/K978C | 25.5 | 30.9±1.19 | 22.0 | 15.4±0.57 | M17M? |

^#^R347 is facing outward in the IWF conformation

^*^D993 is oriented in the outward direction with respective to the channel pore in the IWF conformation
