# Supplementary material for: Transmembrane helical interactions in the CFTR channel pore
Source: PLoS Comput Biol. 2017 Jun 22;13(6):e1005594. doi: 10.1371/journal.pcbi.1005594 (PMC5501672; doi:10.1371/journal.pcbi.1005594)
Supplement: S3 Table — (DOCX) [file pcbi.1005594.s004.docx]

**S3 Table. List of control residue pairs for testing via cysteine cross-linking experiments.**

| Predicted pairs for cross-linking | Cβ-Cβ distance in outward facing model (Å) | | Cβ-Cβ distance in inward facing model (Å) | | Shortest MTS cross-linker for full conversion of c 🡪 x band (Å) |
| --- | --- | --- | --- | --- | --- |
|  | Static | MD | Static | MD |  |
| F342C/T1134C | 17.5 | 9.0±0.36 | 13.0 | 5.0±0.34 | - |
| T339C/T1134C | 18.9 | 12.1±0.27 | 16.9 | 9.8±0.34 | - |
| T338C/E1122C | 32.6 | 23.5±1.10 | 21.3 | 22.0±1.12 | - |
| R334C/V1010C | 24.9 | 20.6±0.64 | 22.7 | 25.0±0.93 | - |
| L188C/V1163C | 22.4 | 18.7±0.84 | 34.4 | 30.7±0.62 | - |
| S185C/V1163C | 24.9 | 21.9±1.70 | 30.2 | 26.3±0.60 | - |

The lack of cross-linking in the above table can be explained as follows: the cross-linking is not seen with T1134 and F342 or T339 since both F342 and T339 orient in the outward direction with respect to the channel pore and therefore two residues in a given pair is not accessible simultaneously. In case of next four pairs, the distance between the pairing residues is longer than the longest MTS reagent used in our experiments as discovered by the model and hence cannot form cross-links. For the R334C/V1010C pair, their exposure is hindered by the presence of another helix.
