# Supplementary material for: Transmembrane helical interactions in the CFTR channel pore
Source: PLoS Comput Biol. 2017 Jun 22;13(6):e1005594. doi: 10.1371/journal.pcbi.1005594 (PMC5501672; doi:10.1371/journal.pcbi.1005594)
Supplement: S5 Table — (DOCX) [file pcbi.1005594.s006.docx]

**S5 Table. Distance comparison between the homology and MD-derived IWF models versus the Cryo-EM structure within the NBD and intracellular regions.**

| Cross-linking pairs | Cβ-Cβ distance in inward facing model (Å) | | |
| --- | --- | --- | --- |
|  | Homology | 5UAK | MD |
| F508C/F1074C | 10.4 | 11.3 | 9.0±1.28 |
| M498C/L1065C | 9.1 | 9.4 | 14.2±0.88 |
| W496C/T1064C | 4.7 | 5.2 | 11.3±1.17 |
| F508C/G1069C | 9.4 | 10.6 | 10.4±0.73 |
| F508C/F1068C | 5.8 | 7.3 | 13.7±0.99 |
| K564C/G1069C | 10.6 | 10.4 | 11.0±0.78 |
| C276C/Q1280C | 6.0 | 7.0 | 5.5±0.70 |
| C276C/K1284C | 4.8 | 8.8 | 9.3±0.60 |
| C276C/Y1307C | 13.4 | 8.9 | 8.1±2.47 |
| G461/S1347 | 31.7 | 26.8 | 36.6±2.47 |
| S549/G1247 | 14.6 | 17.2 | 15.6±1.22 |
