# Supplementary figures and images for: Transmembrane helical interactions in the CFTR channel pore
Source: PLoS Comput Biol. 2017 Jun 22;13(6):e1005594. doi: 10.1371/journal.pcbi.1005594 (PMC5501672; doi:10.1371/journal.pcbi.1005594)

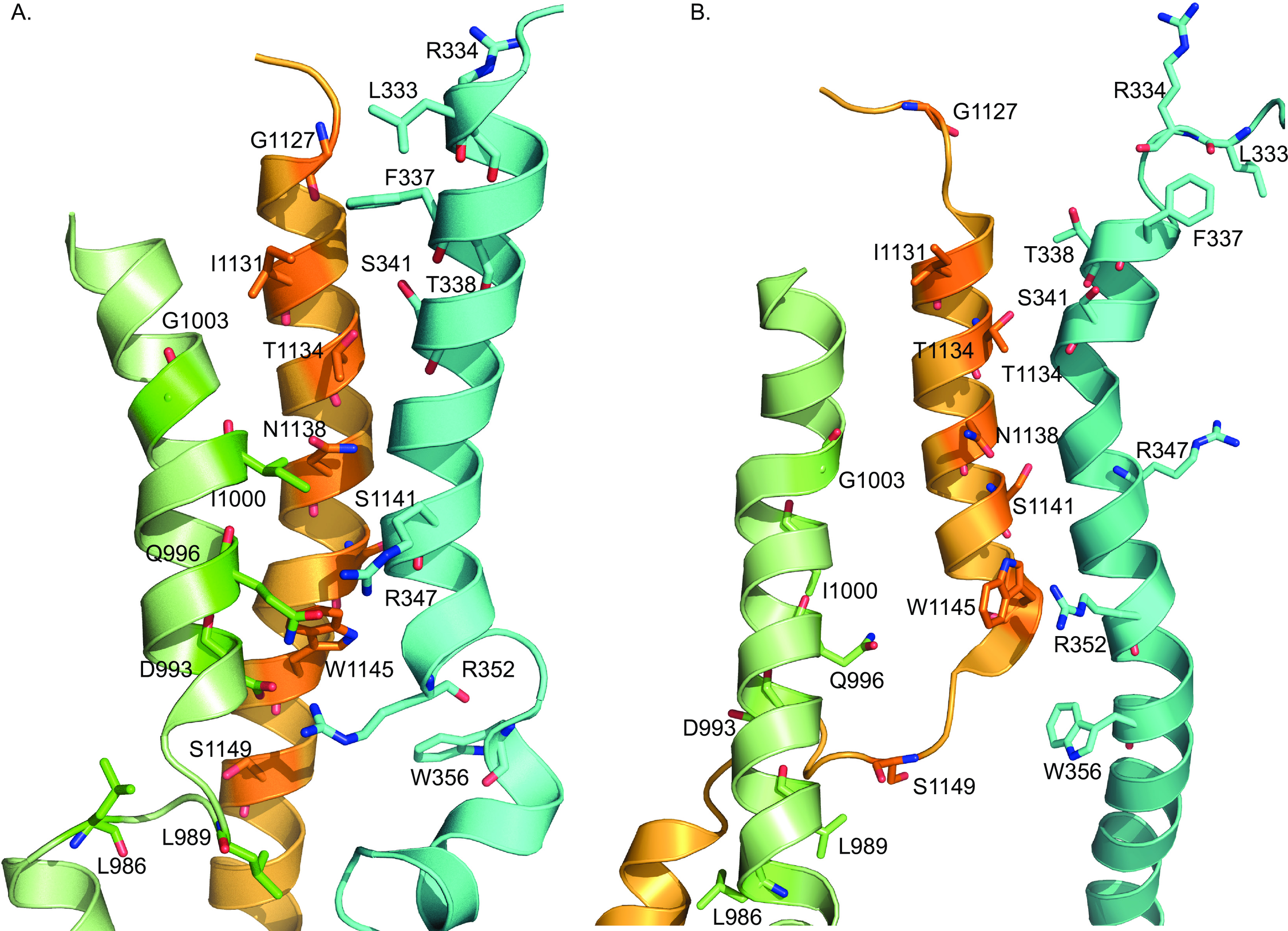

Supplement: S1 Fig — A. outward- and B. inward-facing conformations. The helices are shown with cartoon: Cyan–TMH6, Green–TMH9 and Orange–TMH12. The individual residues are labeled and shown in stick representation. (TIF) [file pcbi.1005594.s007.tif]

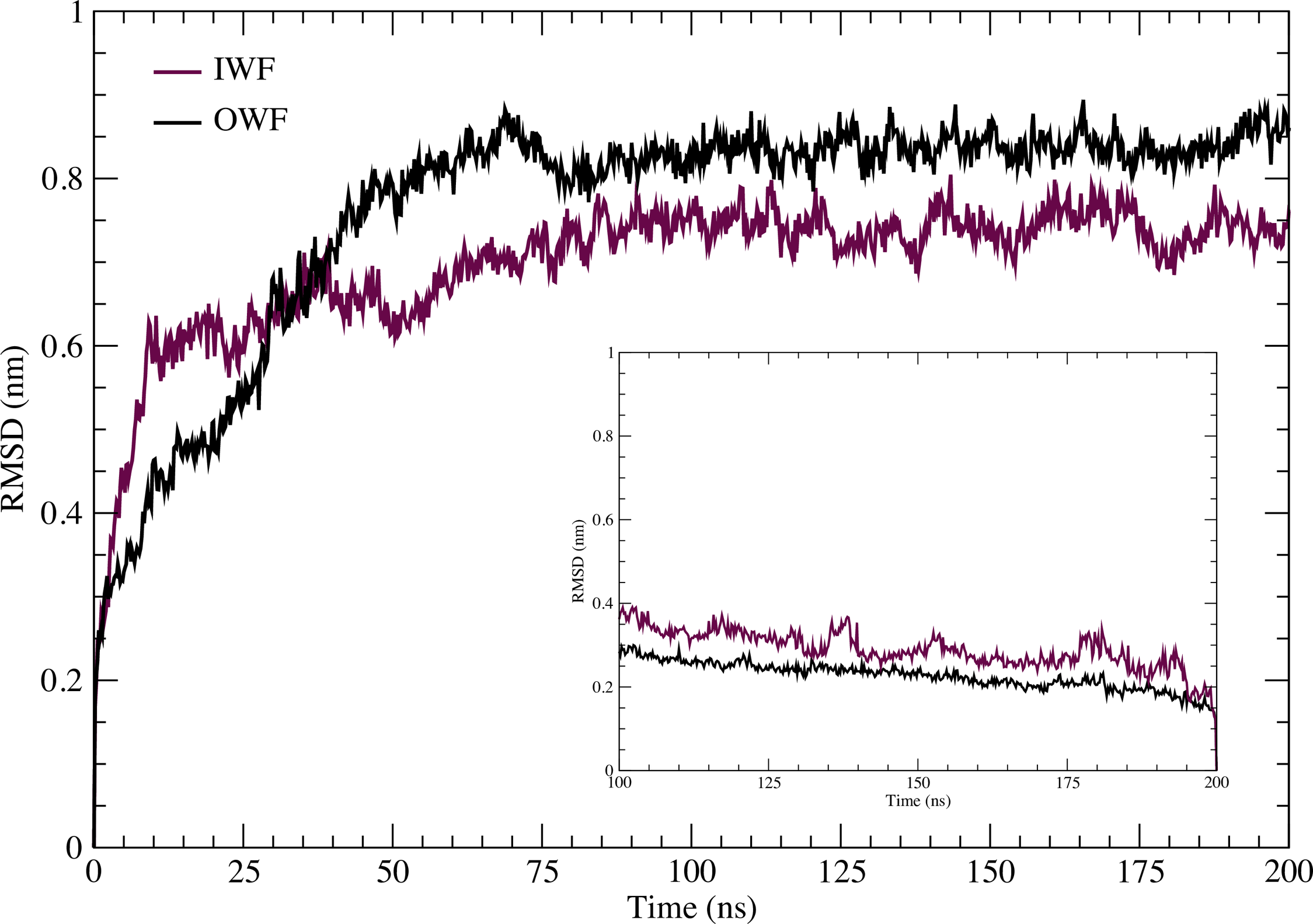

Supplement: S2 Fig — Black curve depicts the RMSD of outward-facing state, and maroon curve shows the RMSD of the inward-facing state with respect to their initial computationally modeled structures. The inset represents the RMSD of protein in OWF (black) and IWF (maroon) states with respect to their final structures obtained at the end of 200 ns simulations. The overall structures do not alter significantly during the last 100 ns production runs as depicted by the RMSD change of ~ 2 Å in both cases. Therefore, the equilibrated structural modes of OWF and IWF configurations can be represented by the 200th ns ones. The representative structures of the OWF conformations from last 100 ns MD runs are depicted in S13 Fig. (TIF) [file pcbi.1005594.s008.tif]

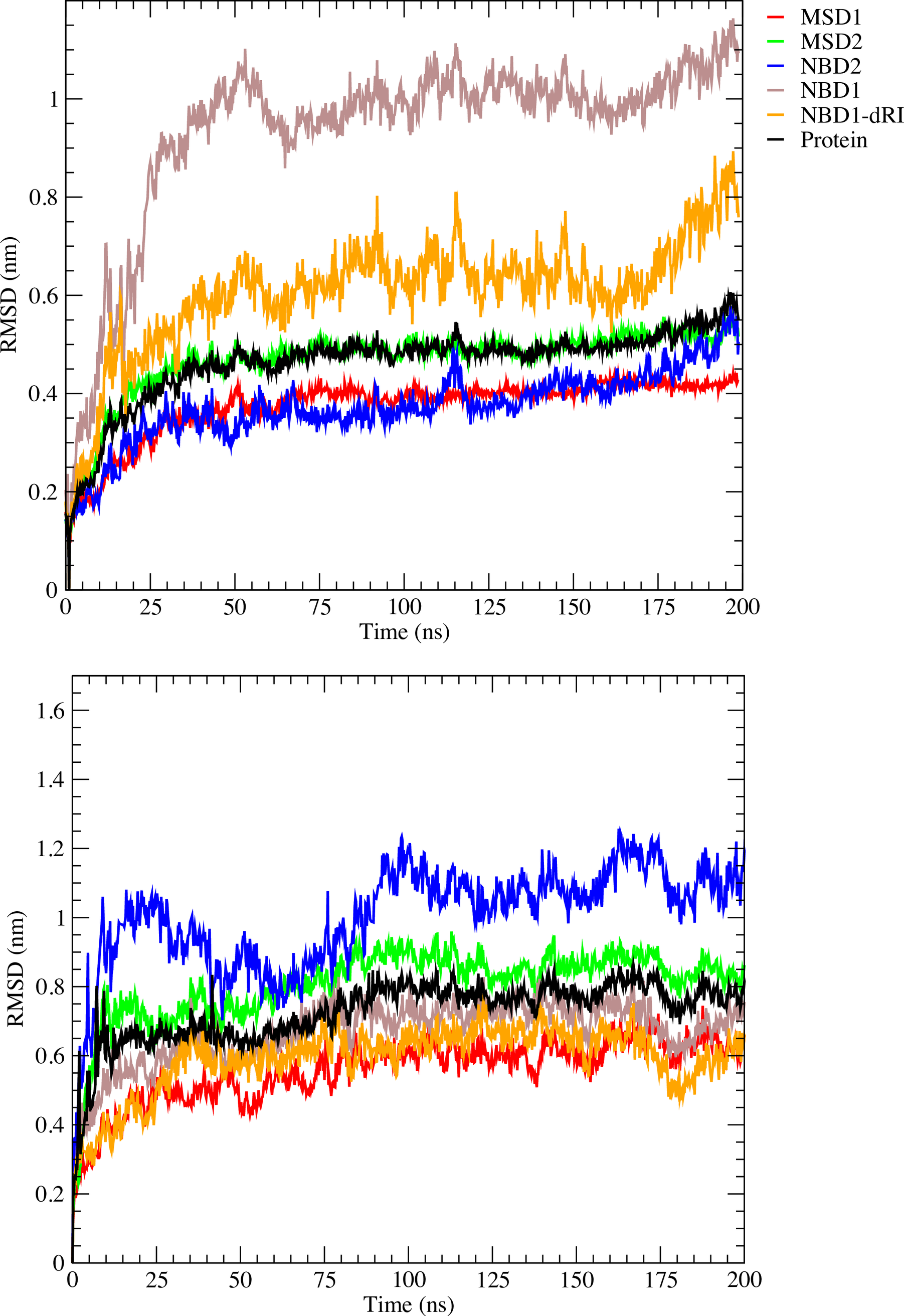

Supplement: S3 Fig — The RMSD of the OWF (upper) and IWF (lower) conformations are evaluated using the entire 200 ns MD trajectory in comparison with the initially computationally built structures before they are subjected to equilibrations. The higher RMSD values in the RMSD of the full-length proteins arise from the disordered regions of NBD1 (OWF state) and NBD2 (IWF state). (TIF) [file pcbi.1005594.s009.tif]

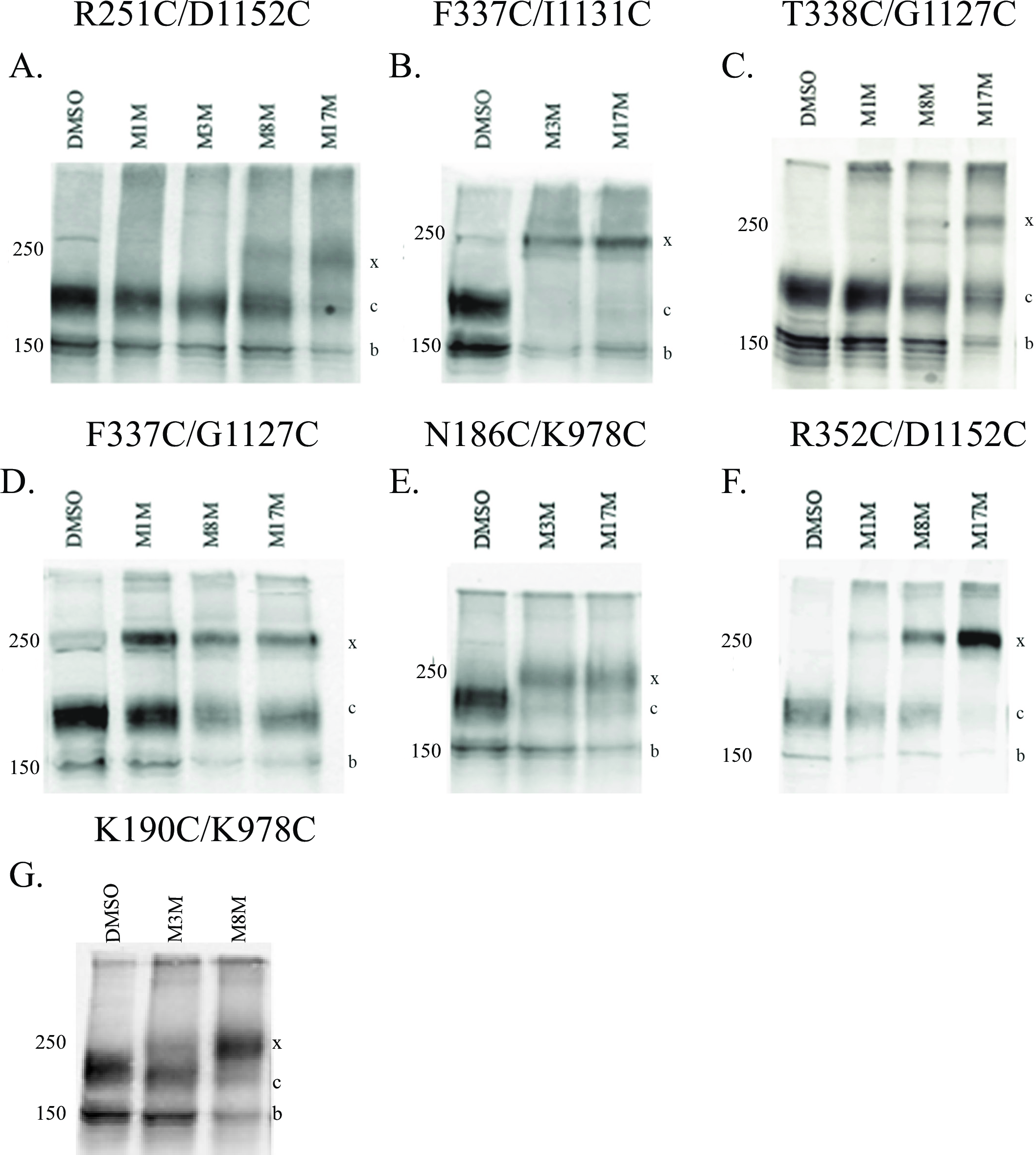

Supplement: S4 Fig — Among the tested 29 pairs, 23 pairs show cross-links upon treatment with MTS reagents of various lengths. Shown here are 7 pairs of cross-links. Immature core-glycosylated CFTR is marked as band b; mature complex-glycosylated CFTR as band c; band x represents cross-linked mature protein. Overall correspondence between the relative distances between all the residue pairs retrieved from cross-linking experiments and the newly refined outward facing CFTR structure is 100% (refer to S2 Table). Note that, the putative CFTR pore spans the entire pore region including the extracellular, membraneous and intracellular regions. (TIF) [file pcbi.1005594.s010.tif]

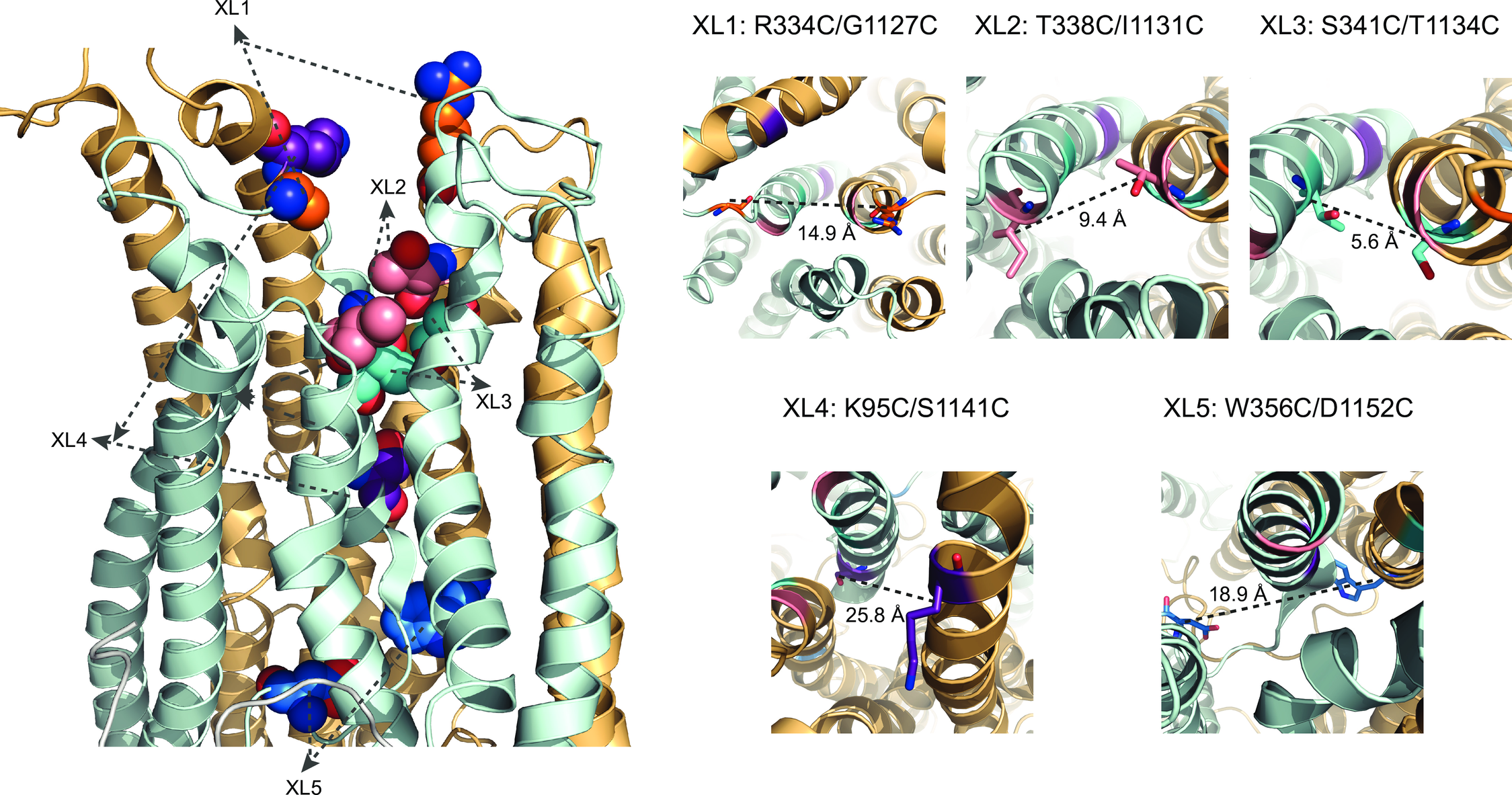

Supplement: S5 Fig — Left: refined inward facing state of CFTR model exhibiting examples of residue pairs XL1, XL2, XL3, XL4 and XL5 (spheres) used for testing the reliability of the CFTR pore. The inward facing CFTR structure presented here is obtained from 200 ns MD simulation. Right: the average distances between each residue pairs. This figure is related to Fig 3 and the corresponding western blots are exhibited in the same. (TIF) [file pcbi.1005594.s011.tif]

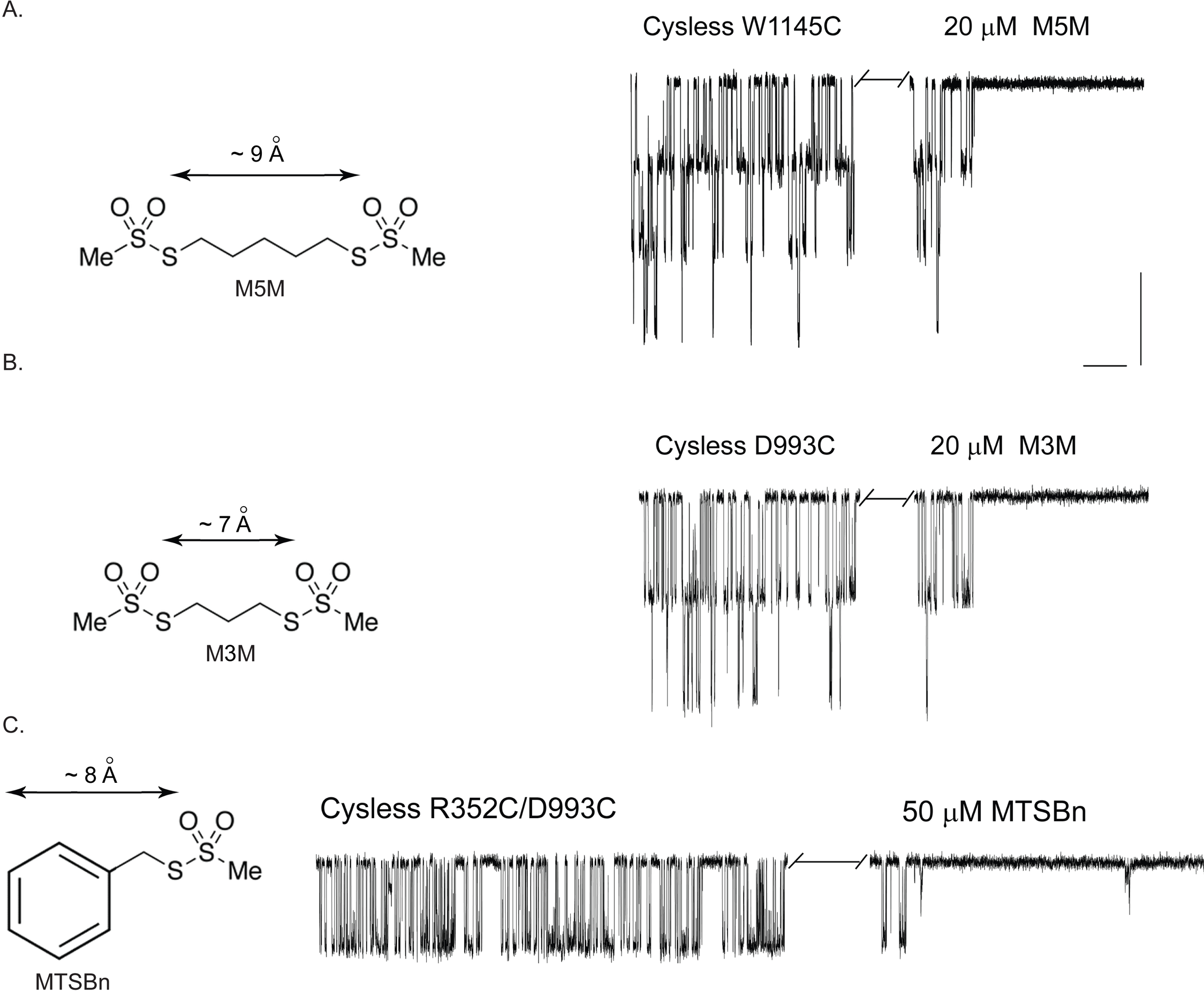

Supplement: S6 Fig — (A) Blockage of W1145C channel by 20 μM M5M. Three Cys-less W1145C channel recordings with the wild type CFTR ion pore conductance of 12.3 pS is shown. The technical gap in the recording is shown as a bar and represents a 2 minutes interval needed for 20 μM M5M MTS reagent application to the cis side of the bilayer. All three channels were converted to a nonconductive state shortly after recording resumed. (B) Blockage of D993C channel by 20 μM M5M. Two channel recordings of Cys-less D993C construct with the pore conductance of 14.5 pS. The negative charge removal increases pore conductance in comparison with the wild type CFTR only slightly. The gap in the recording is shown as a bar and represents about 2 minutes time interval needed for the 20 μM M3M application at the cis side. Both channels were turned into the nonconductive state shortly after recording resumed. (C) Blockage of R352C/D993C channel by 50 μM MTSBn. Vertical scale bar of 1pA and horizontal scale bar of 10s are common for all traces. The chemical structures of M5M, M3M and MTSBn reagents are shown on the left side of each tracing. (TIF) [file pcbi.1005594.s012.tif]

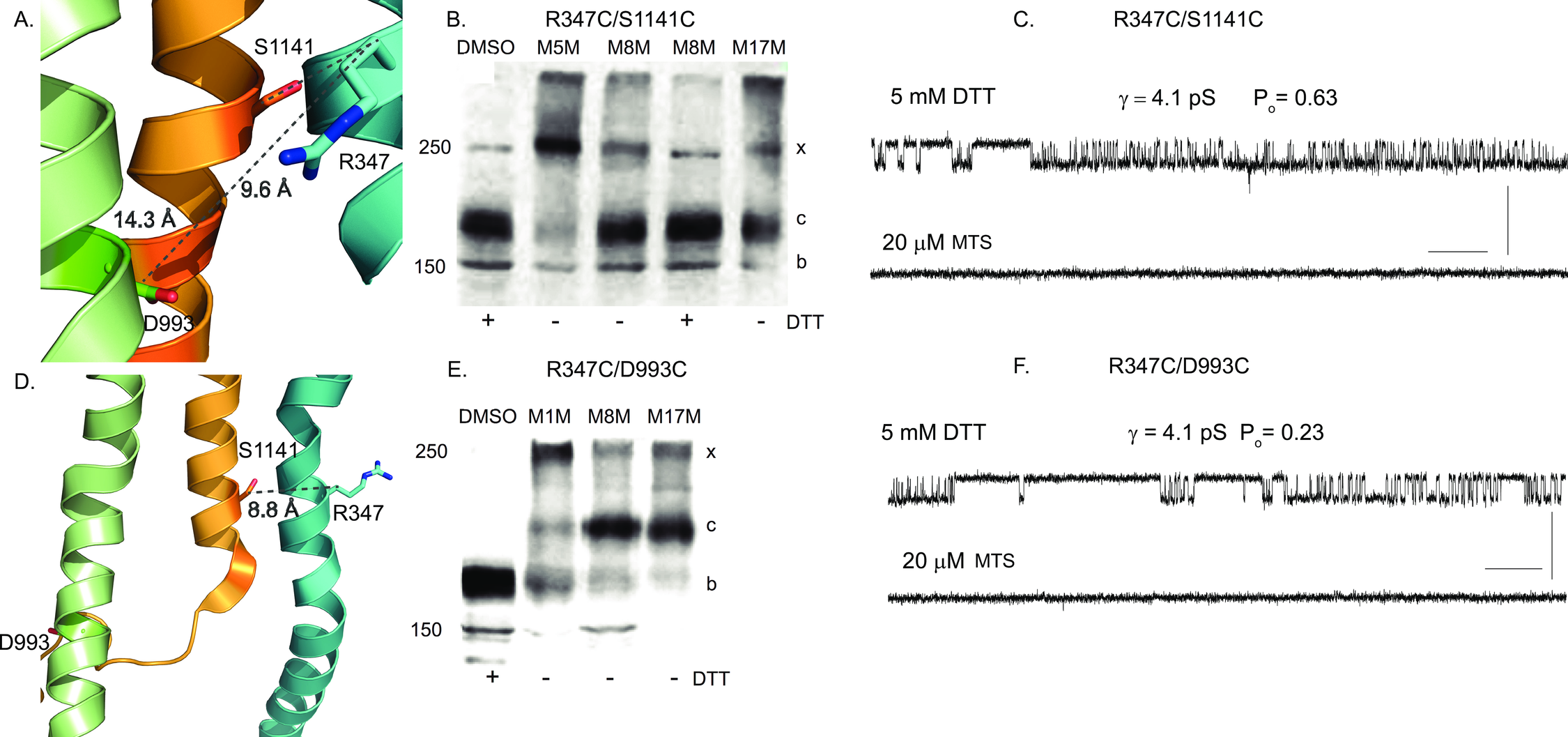

Supplement: S7 Fig — (A) Relative positions of R347 in TMH6, D993 in TMH9, S1141 in TMH12 in outward facing state. (B) Western blot of untreated Cys-less CFTR R347C/S1141C constructs (lane 1) and treated with 20 μM of MTS cross linkers of different length (lanes 2–4). No change in mature band position of untreated sample (lane 1) suggests that no tight contact exists between cysteines at these positions. Thus, the inter-residue distance fluctuates significantly. (C) 10 minute single channel recording of Cys-less R347C/S1141C with γ = 4.1 pS under native conditions (reduction by 5 mM DTT) without any treatments with other thiol reagents. No opening is observed during the recording period after treatment with 20 μM M5M MTS reagent (lower tracing). (D) Relative positions of R347 in TMH6, D993 in TMH9, S1141 in TMH12 in inward facing state. (E) Western Blot of untreated Cys-less CFTR R347C/D993C constructs (lane 1) and treated by 20 μM of MTS cross linkers of different lengths (lanes 2, 3, and 4). No change in mature band position without treatment (lane 1) indicates that there is no spontaneous S-S bond formation between R347C and D993C while only M17M causes a strong shift of mature c-band to the x-band. (F) Single channel recordings of R347C/D993C with γ = 4.1 pS under the reducing conditions induced by 5 mM DTT. Channel conductance is lost as a result of cross-linking by 20 μM M17M (lower tracing). (TIF) [file pcbi.1005594.s013.tif]

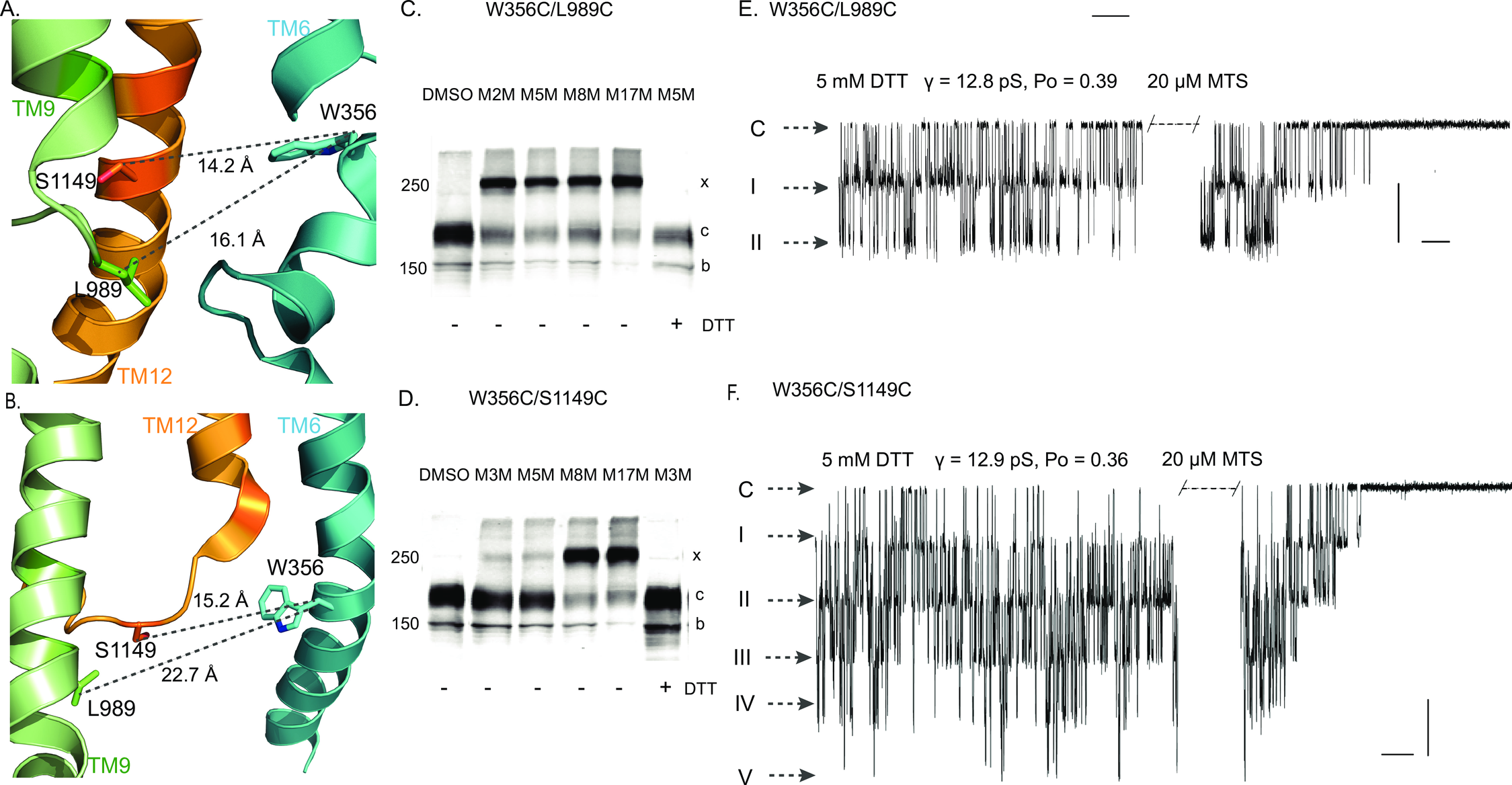

Supplement: S8 Fig — (A) Locations of W356, L989 and S1149 residues residing in TMHs 3, 9 and 12, respectively in A) outward facing state. (B) Western blot of Cys-less CFTR W356C/L989C before (lane 1) and after treatment with MTS (20 μM) reagents (lanes 2–6). No change in c band position without treatment suggests that no tight contact exists between cysteines at these positions. Strong shift of c to x band position is observed after treatment by cross linkers M2M through M17M. (C) Recordings of two independent Cys-less W356C/L989C CFTR ion channels with γ = 12.8 pS are shown on left: these independent channels are labeled as I, and II; channel closing is labeled with C. Recording of the same membrane after adding 20 μM M5M cross-linker at the “cis” side is shown on right. A 20s interruption in the recording used for cross-linker application and stirring is shown by the dotted line. (D) Positions of W356, L989 and S1149 in the inward facing state. E) Western blot of Cys-less CFTR W356C/S1149C before (lane 1) and after treatment with 20 μM MTS cross-linkers (lanes 2–6). (F) Recording of five independent Cys-less W356C/S1149C CFTR channels with γ = 12.9 pS (shown on left): the five channels are labeled as I, II, III, IV and V; channel closing is indicated by C. Recording of the same multi-channel membrane after application of 20 μM M3M at the “cis” side is on the right. There is a 20s interruption in the recording for cross-linker application, the dashed line shows stirring. Vertical (X) and horizontal (Y) axes scale bars represent 10s and 1 pA, respectively. (TIF) [file pcbi.1005594.s014.tif]

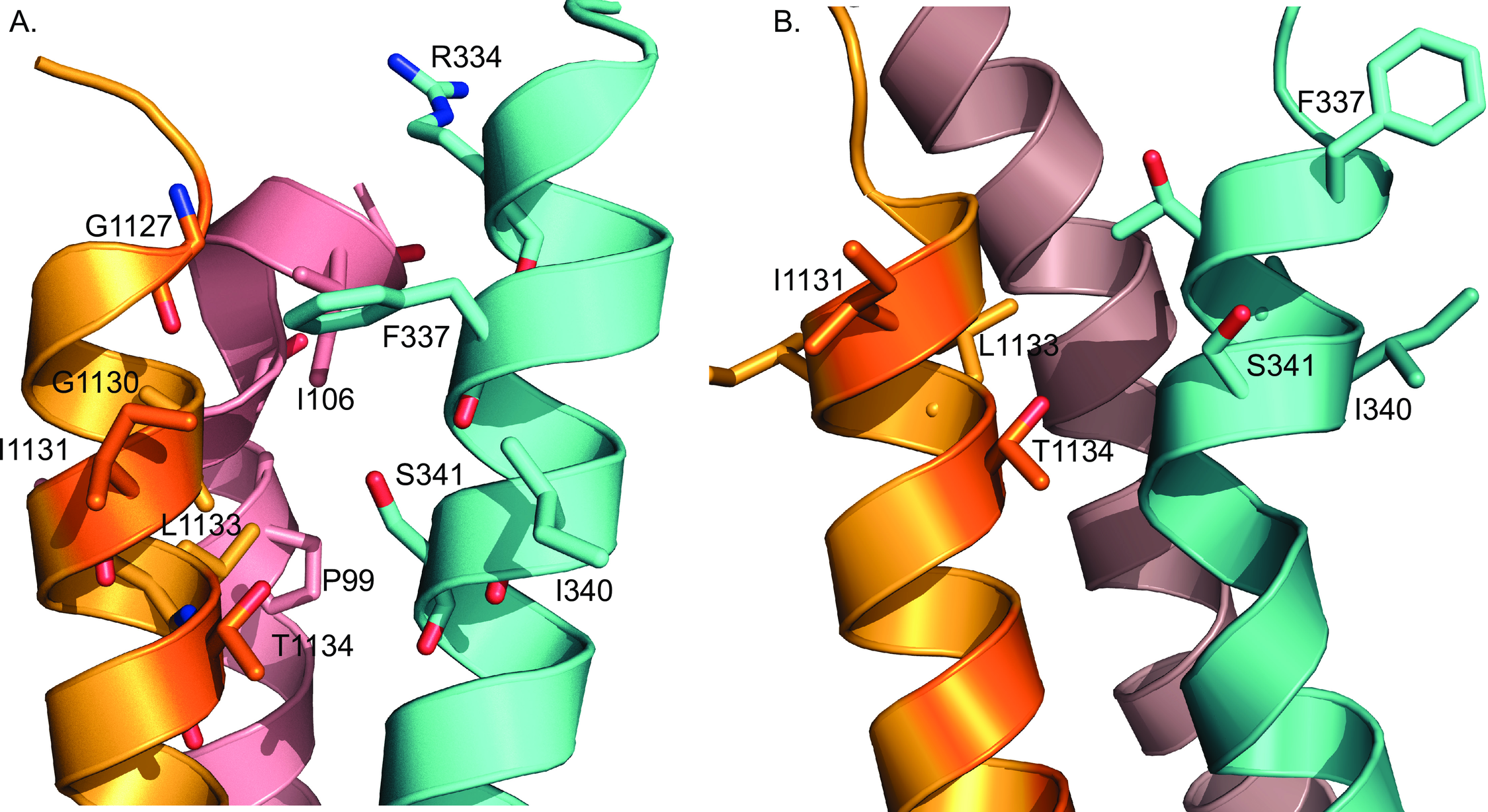

Supplement: S9 Fig — (A) outward- and (B) inward-facing conformations. The TMHs 1 (pink), 6 (cyan), and 12 (orange) are color-coded. These structures are the final conformations of the CFTR channels after 200 ns molecular dynamics simulations. (TIF) [file pcbi.1005594.s015.tif]

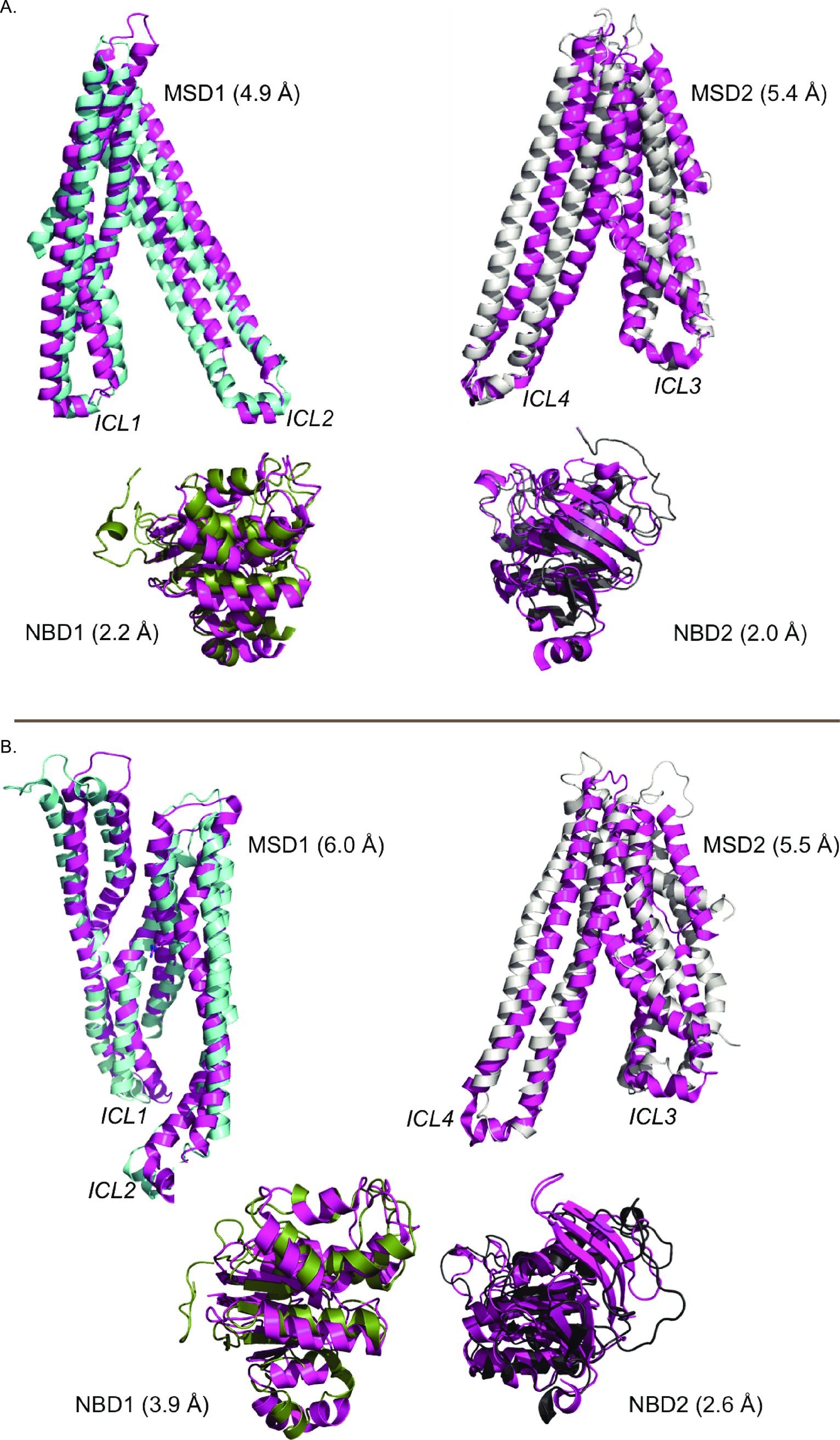

Supplement: S10 Fig — A. homology model based CFTR structure versus 5UAK, and B. channel structure obtained after 200 ns MD equilibration versus 5UAK. The magenta colored structure represents cryo-EM model and our MSD1, MSD2, NBD1 and NBD2 structures are depicted with cyan, grey-white, green and dark gray colors. (TIF) [file pcbi.1005594.s016.tif]

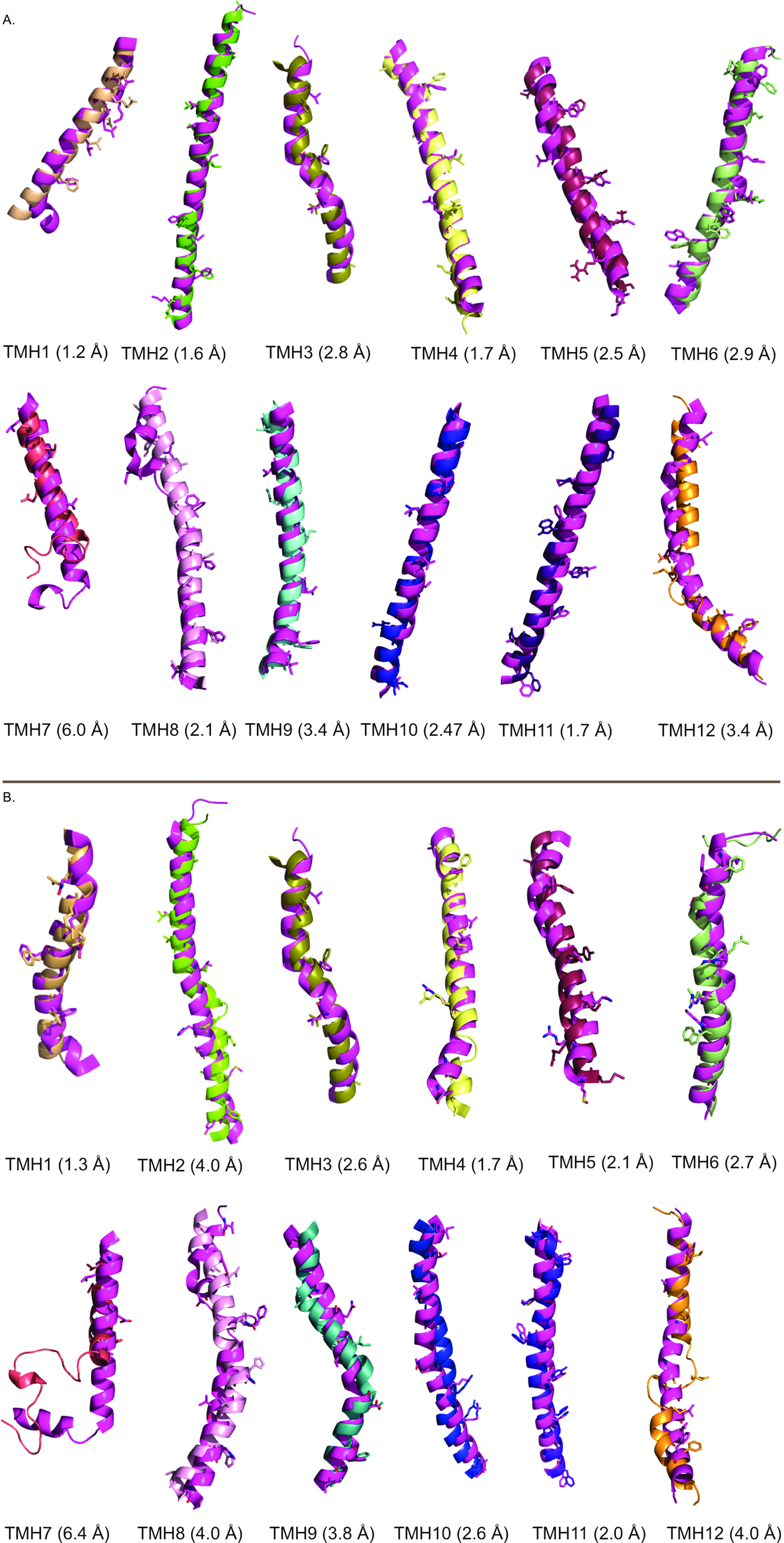

Supplement: S11 Fig — A. The homology model based structure versus 5UAK and B. CFTR structure at the end of 200 ns MD equilibration versus 5UAK. The magenta colored helices represent cryo-EM model and our TMHs are depicted with following colors: TMH1 –wheat, TMH2 –green, TMH3 –olive, TMH4 –yellow, TMH5 –salmon, TMH6 –sea green, TMH7 –raspberry, TMH8 –pink, TMH9 –cyan, TMH10 –blue, TMH11 –purple, TMH12—orange. (TIF) [file pcbi.1005594.s017.tif]

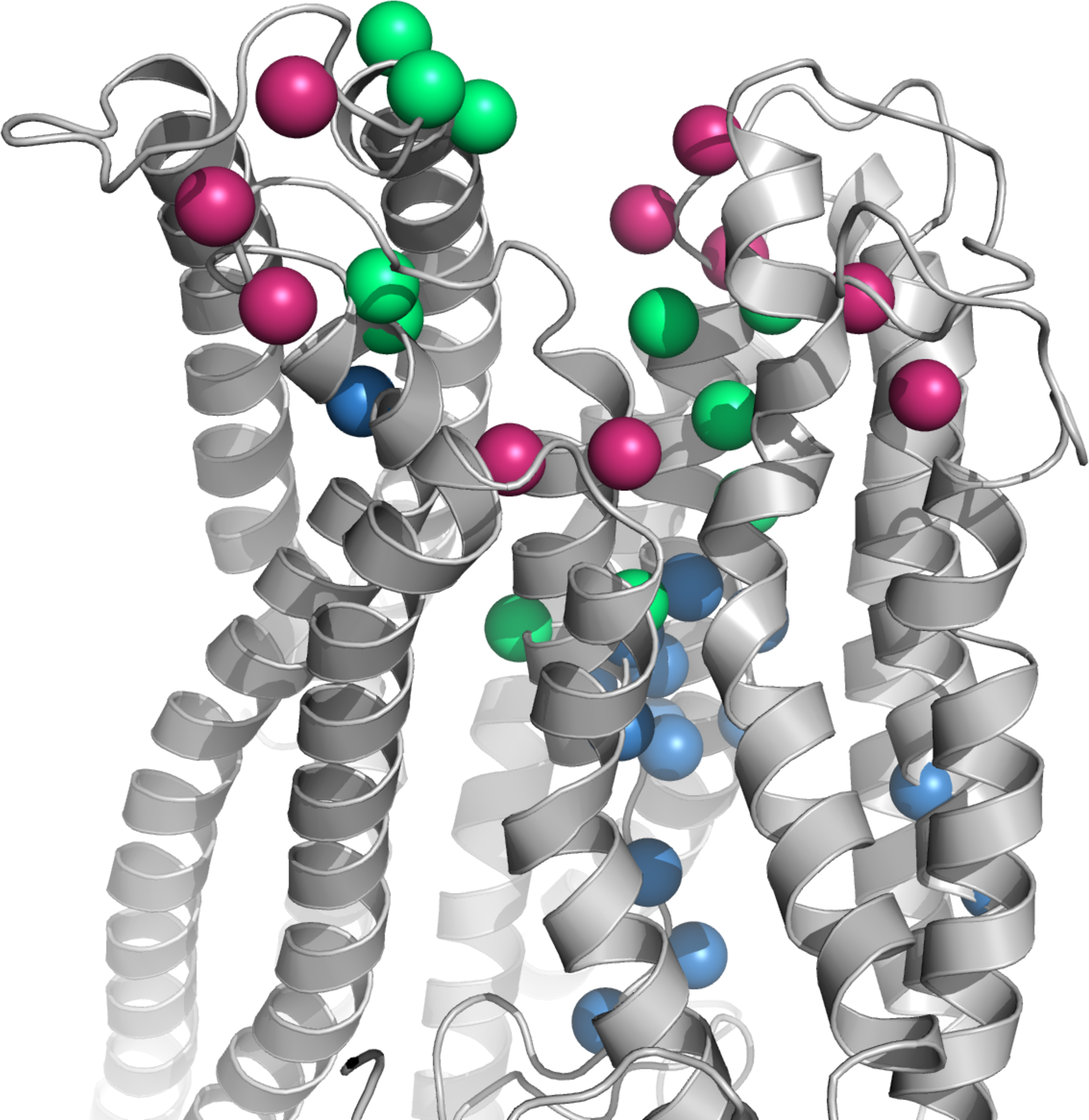

Supplement: S12 Fig — Residues accessible from the intracellular side of the cell: S307, F311, V345, M348, A349, R352, Q353, T1112, S1141, T1142, Q1144, W1145, V1147, N1148, S1149 (Cβ atoms are presented using blue spheres); residues accessible from the extracellular side of the cell: R104, L323, A326, R334, K335, I336, I1121, T1122, I1131, and I1132 (Cβ atoms are presented using magenta spheres); and the pore lining residues: Q98, P99, L102, F337, T338, S341, I344, T1115, S1118, N1138, and M1140 (Cβ atoms are presented using green spheres). (TIF) [file pcbi.1005594.s018.tif]

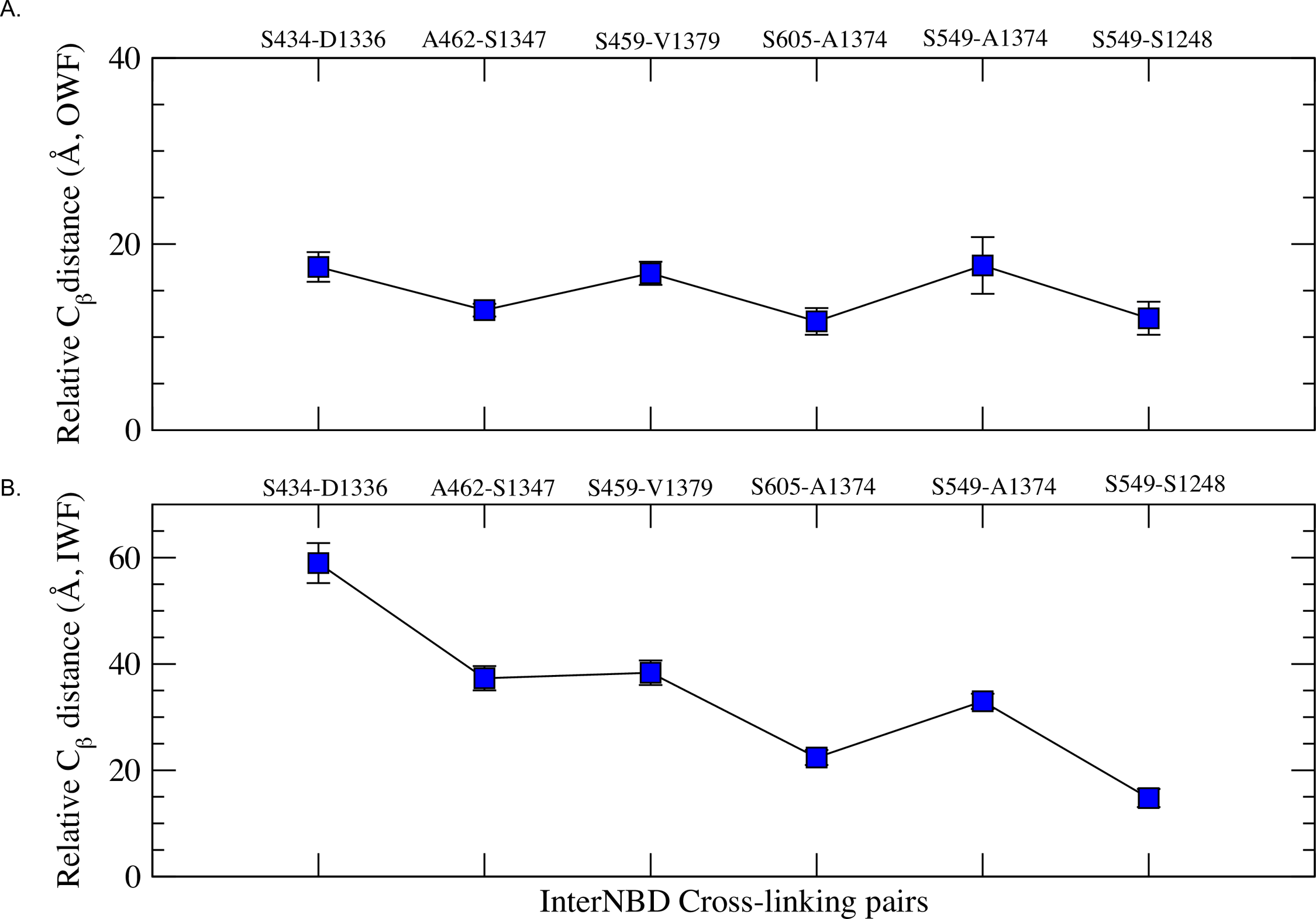

Supplement: S13 Fig — A. OWF structure (upper panel) and B. IWF structure. The average distances and the standard deviations are quantified by sampling 200 ns MD trajectory. (TIF) [file pcbi.1005594.s019.tif]

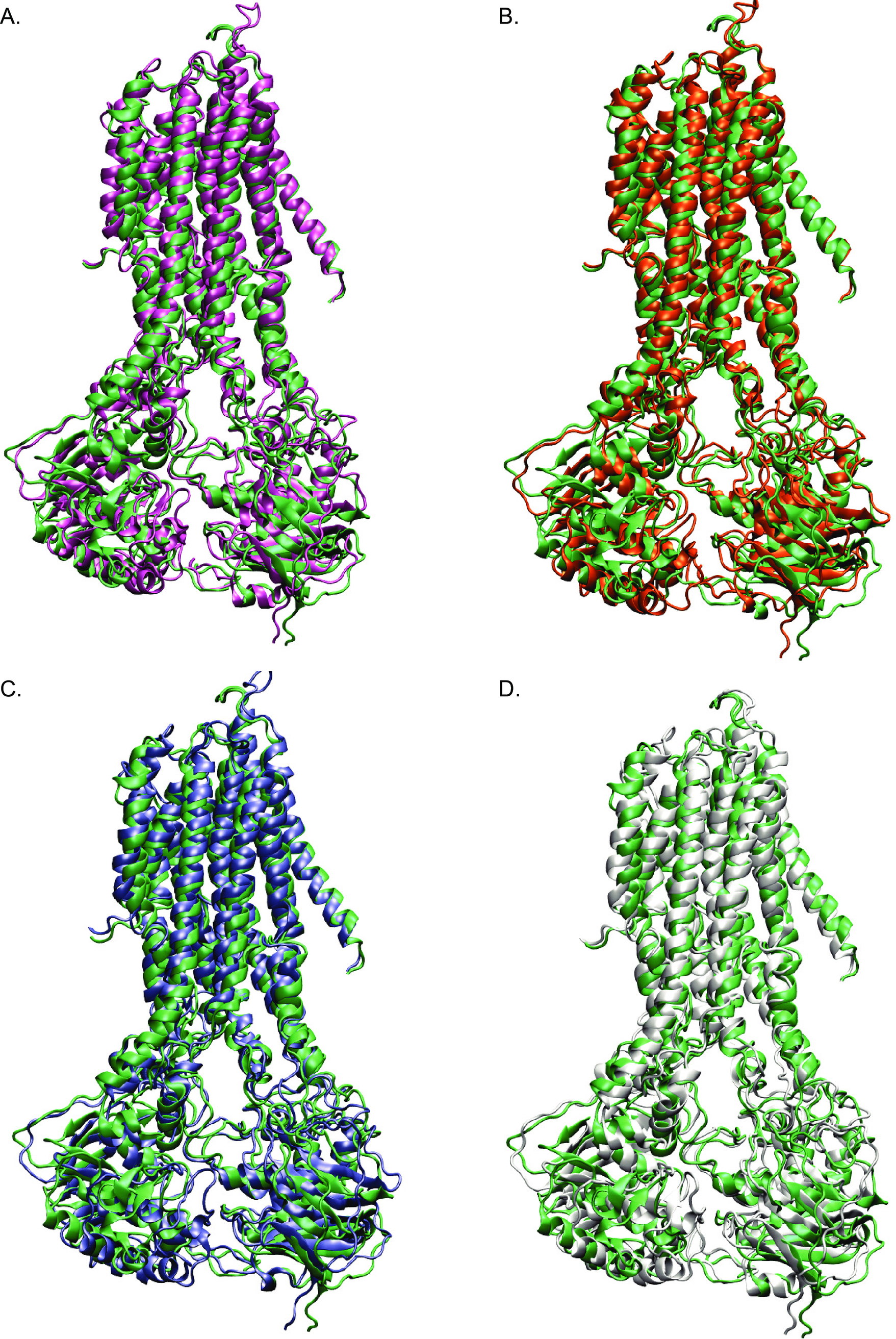

Supplement: S14 Fig — Representative centroid structure of (A) cluster 5 (green) versus 1 (pink) (B) cluster 5 (green) versus 2 (orange) (C) cluster 5 (green) versus 3 (ice blue) and (D) cluster 5 (green) versus 4 (white). The representative structures from five different clusters are very similar suggesting that the transition between different conductive and non-conductive states is not attained during the span (~ 200 ns) of the simulation. Longer simulations are required for studying channel gating and associated alternate conformations of the channel. In both cases, the clustering is obtained by isolating structures within a RMSD of ~ 2 Å of one another. Note that only backbone atoms are considered. The representative structure presented here is simply the centroid of each cluster. Cluster 5 is taken as the reference model as it has the highest population. Clustering analyses yields five clusters from the entire production run based on our clustering criterion. (TIF) [file pcbi.1005594.s020.tif]

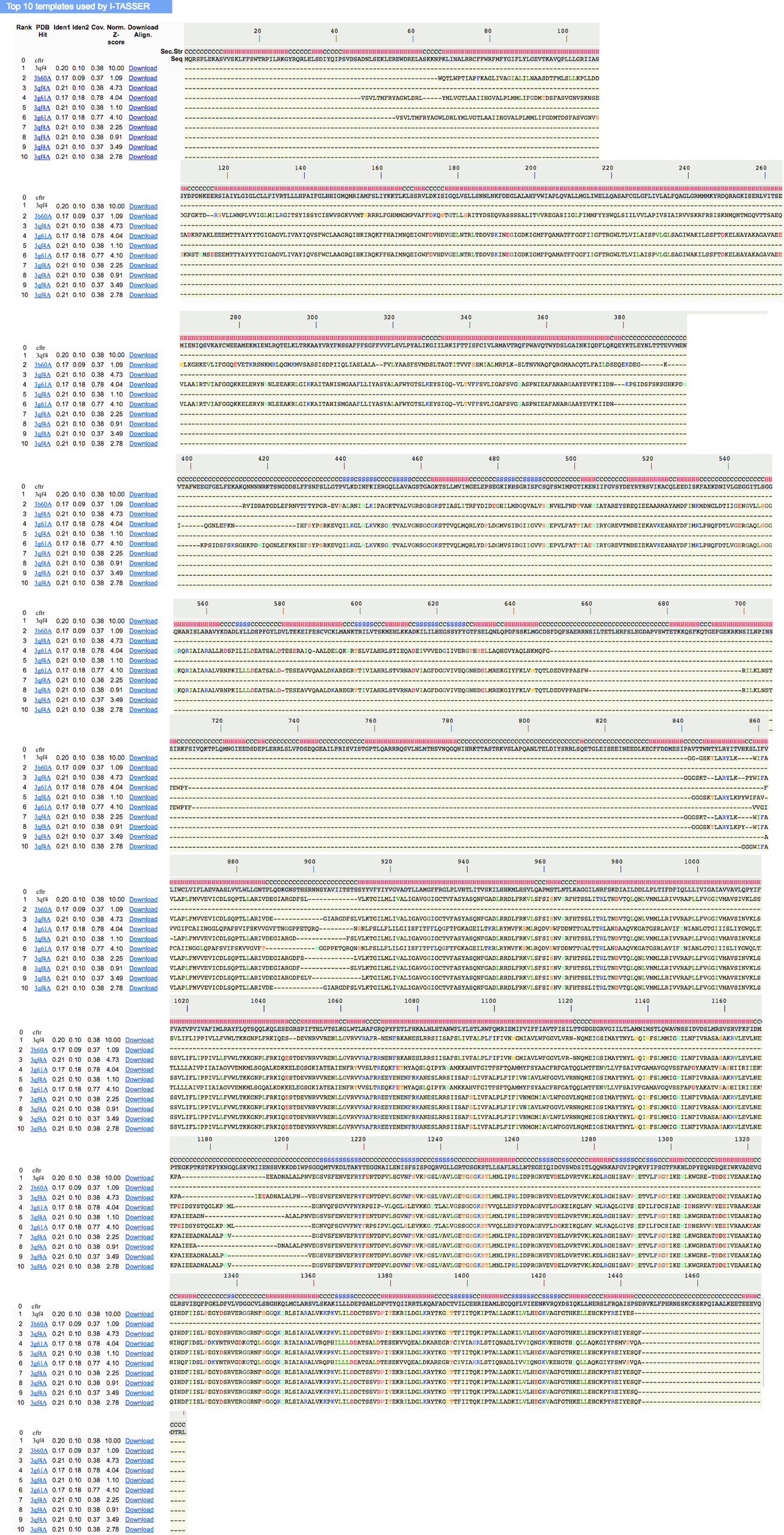

Supplement: S15 Fig — The sequence alignment of CFTR versus the structural templates applied in model building is presented. (TIF) [file pcbi.1005594.s021.tif]
